# Use of wild vertebrates for consumption and bushmeat trade in Brazil: a review

**DOI:** 10.1186/s13002-023-00628-x

**Published:** 2023-12-19

**Authors:** José Augusto Aragão Silva, Leonardo Moura dos Santos Soares, Felipe Silva Ferreira, André Bastos da Silva, Wedson Medeiros Silva Souto

**Affiliations:** 1 Development and Environment Graduated Program, Federal University of Piauí (UFPI), Teresina, Piauí CEP: 64049-550 Brazil; 2 State University of Maranhão (UEMA), Coelho Neto, MA CEP: 65620-000 Brazil; 3 Graduated Program in Health and Biological Sciences, Federal University of Vale de São Francisco (UNIVASF), Petrolina, PE CEP: 56304-917 Brazil

**Keywords:** Wildlife, Food consumption, Trade chain, Ethnozoology, Conservation

## Abstract

**Background:**

Bushmeat is a resource exploited by thousands of people around the world, especially in tropical and neotropical regions, constituting an important source of protein and income. But what is known, so far, about the consumption and trade of wild vertebrate meat (hereinafter “bushmeat”) in a megadiverse country like Brazil? This question was answered through a systematic survey of publications on the consumption and trade of wild vertebrate meat made in Brazil between 2011 and 2021.

**Methods:**

We selected 63 scientific articles available on “Google Scholar,” “Science Direct,” “Scopus,” “ Web of Science” and “Portal de Periódico da CAPES.” The articles were categorized as: exclusive to (1) consumption or (2) bushmeat trade, totals of 54 and three articles, respectively; both (3) consumption and trade bushmeat, totaling six articles. We applied a nonparametric Spearman's correlation analysis to verify the association between the number of papers and the species richness of wild vertebrates cited for consumption by Brazilian state.

**Results:**

The results revealed that the publications were concentrated in the Northeast (36), North (26) and Southeast (1) regions, distributed across 16 states of the federation. These data reinforce the need for more researches in states and other regions of the country. Our research hypothesis was confirmed, since the richness of species cited for meat consumption was positively associated with the amount of work carried out by the states of the federation. We identified a total of 321 species of wild vertebrates mentioned in the categories involving the consumption of bushmeat. We had a greater bird species richness mentioned for consumption (170) to the detriment of mammals (107), reptiles (40) and amphibians (4). Furthermore, in the articles involving the bushmeat trade categories we had 57 species of vertebrates mentioned, with mammals being the most representative in terms of species richness (29), to the detriment of birds (20) and reptiles (8). These data reinforce that birds and mammals have been the groups most used both for consumption and trade in bushmeat in the country's regions, and it is necessary to mitigate the hunting exploitation of these groups. We recorded that socioeconomic, biological, environmental and sociocultural factors were the most cited predictors of the consumption and trade of bushmeat in the articles. We identified that the bushmeat trade chain is dynamic and ramified, made up of several actors, including specialized and diversified hunters, intermediaries, market sellers, market vendors, restaurant owners and final customers. Public markets and open-air fairs were the most cited places for buying and selling wild meat in commerce.

**Conclusions:**

In general, our results indicate that we have made significant advances in publications on the consumption and trade of bushmeat in Brazil over the last few years. However, we highlight the need to better understand the patterns of consumption and trade of bushmeat in different regions of the country, as well as the factors associated with the dynamics of the trade chain and uses of wildlife by local communities. We emphasized that a multidimensional understanding of hunting activities is important to face socio-ecological problems and improve the conservation of target species which have continually been explored for uses by populations in different regions of the world.

**Supplementary Information:**

The online version contains supplementary material available at 10.1186/s13002-023-00628-x.

## Background

Historically, hunting wild animals played a significant role in the evolution of humanity and the formation of cultures around the world [1, 2]. In the current context, hunting still plays an important role in the survival of human populations, which use wild fauna species as a source of food, medicinal use, commercial, ornaments, clothing, magical-religious, *pets*, social relationships in general, among other purposes [2–7].

The different uses of wild fauna by human societies have encouraged hunting practice, a secular activity of relevant socioeconomic and cultural importance, especially for people from tropical and neotropical forests [8, 9]. Hunting is a practice considered successful in the exploitation of faunal resources; bushmeat, for example, is an important faunal resource used as ensuring food safety and income generation thousands of populations in tropical regions of the world, mainly in Africa, Asia and Latin America [10–13]. In the current context, the use of wild animals for food consumption continues to contribute to the diversification of the diet throughout the world [14–16]. For example, a study by Nielsen et al. [11] estimated that between 230 and 833 million people living in the tropics depend on the meat of wild vertebrates (amphibians, reptiles, birds and mammals) as a source of protein. In urban regions of Central Africa, for example, it is estimated that populations consume more than 4.5 million tons of bushmeat annually in the Congo basin [17, 18].

However, in recent decades, the use of wild animals for their meat has gone from just a source of food and income for rural tropical populations, to a commodity exploited for profit to supply urban areas [19, 20]. This increase of trade urban has increased the prices of bushmeat products, intensified bushmeat harvests and affected hunting patterns and wildlife utilization in the tropics [13, 21, 22]. For example, studies estimate that around 100 million wild animals are traded annually in the tropics, comprising around 6000 species, with an annual global value of US$7 to US$23 billion [19, 20].

The increase in the exploitation of wild fauna by populations in the tropics, on the other hand, has made the levels of use of wild animals unsustainable, directly impacting the conservation of biodiversity [23, 24]. The impacts of unsustainable hunting for wild animal meat threaten the survival of several species that live in the world's tropical forests, mainly primates, large ungulates (such as tapirs and peccaries) and large birds such as curassows, in addition to ecological consequences [23, 25–28].

The practice of hunting in Brazil, despite the context of illegality [29], is permitted only for indigenous peoples and local communities in case of hunger. However, bushmeat hunting continues to be practiced in all regions and biomes [30]. The persistence of hunting in the country has been associated with different socioeconomic, political and cultural contexts, with bushmeat having nutritional importance and generating income for income for several rural and urban populations [31–36]. In the current context, dependence on bushmeat as a nutritional, economic and cultural component for subsistence is still a prevalent reality in many communities in rural and urban areas of Brazil [37–39]. A study by Nyaki et al. [22], for example, estimated that approximately 10 thousand tons of meat from hunting is consumed annually by urban populations residing in the central Brazilian Amazon.

Despite the wide dissemination of hunting and uses of wild fauna and the importance of exploring these resources in Brazil, studies that address hunting are incipient when compared to other parts of the tropics [9, 40, 41]. Most ethnozoological publications carried out in the country have focused on the use of wild animals as a source of meat, traditional medicine and *pets*, mainly due to the greater wealth of species exploited and commercialized for these purposes [7, 14, 42, 43]. Nonetheless, there are several gaps in information about hunting and uses of fauna that need to be filled, mainly about the richness of species exploited for consumption and trade in bushmeat in regions of Brazil [41].

Ethnozoological studies can contribute to the implementation of public policies aimed at the management and conservation of wild fauna [6]. It this context the present evaluated the current situation of publications on the consumption and trade of wild vertebrate meat in the regions of Brazil from 2011 to 2021. More specifically, we sought to identify the richness of animal species exploited for consumption and trade bushmeat in the country's regions and verifying the factors associated with the consumption and trade chain. We tested the hypothesis that the richness of species cited for bushmeat consumption would be greater in the states of the federation with a greater number of selected consumption publications.

## Methods

### Data collection

We delimited our review to scientific articles on the theme of consumption and trade of wild vertebrate meat carried out in the federative units of Brazil between the years 2011 and 2021. We searched the following databases: Google Academic, Science Direct, Scopus, Web of Science and Portal de Periódico da CAPES (Coordenação de Aperfeiçoamento de Pessoal de Nível Superior). To do this, we used combinations of carefully selected keywords in Portuguese, English and Spanish (Table 1).

**Table 1:** Results of searches and combinations of keywords applied databases in the period of 2011 to 2021 in Brazil

Keywords	Results GA	Articles selected	Results PPC	Articles selected	Results SC	Articles selected	Results SD	Articles selected	Results WS	Articles selected
*Keywords in Portuguese*										
“Carne de caça” + consumo + vertebrados + Brasil	160	09	28	0	–	–	–	–	–	–
“Carne de caça” + caça + vida selvagem + Brasil	166	03	47	0	–	–	–	–	–	–
“Carne de caça” + comércio + ilegal + Brasil	561	0	12	0	–	–	–	–	–	–
“Carne de caça” + urbano + rural + Brasil	872	0	13	0	–	–	–	–	–	–
“Carne de caça” + colheita + animais silvestres + Brasil	369	0	90	0	–	–	–	–	–	–
Keywords in English										
“Bushmeat” + consumption + vertebrates + Brazil	248	17	238	0	41	0	73	0	40	0
“Bushmeat” + hunting + wildlife + Brazil	717	07	171	0	25	0	75	0	14	0
“Bushmeat” + trade + illegal + Brazil	528	0	180	0	13	0	122	0	11	0
“Bushmeat” + urban + rural + Brazil	464	0	185	0	39	0	14	0	16	0
“Bushmeat” + harvest + wild animales + Brazil	164	0	158	0	52	0	60	0	04	0
*Keywords in Spanish*										
“Carne de monte” + consumo + vertebrados + Brasil	97	0	24	0	–	–	–	–	–	–
“Carne de monte” + caza + fauna salvaje + Brasil	420	0	21	0	–	–	–	–	–	–
“Carne de monte” + comercio + ilegal + Brasil	397	0	12	0	–	–	–	–	–	–
“Carne de monte” + urbano + rural + Brasil	351	0	19	0	–	–	–	–	–	–
“Carne de monte” + cosecha + animales silvestres + Brasil	236	0	10	0	–	–	–	–	–	–
Total	5.750	36	1.208	0	170	0	344	0	85	0

We initially selected the scientific publications by reading and analyzing the titles and abstracts, applying the following inclusion criteria: (1) mention of the use of wild vertebrates for consumption and the hunting meat trade in Brazilian federative units; (2) mention of factors driving the consumption of wildmeat vertebrate; (3) mention of aspects of the bushmeat trade chain. In addition, we selected additional papers found in the reference lists of the articles found in the databases and that met the inclusion criteria.

In the second stage all articles initially selected were read in full, and we excluded articles based on the following criteria: (1) use of wildmeat vertebrate only as a source of nutritional value and its relationship with human health; (2) use of bushmeat and the transmission of pathogens and zoonotic diseases; (3) use of bushmeat for zootechnical purposes and associated products; (4) consumption and trade in the meat of aquatic animals (fish, mollusks, crustaceans) and other groups of invertebrates.

We exclude also duplicate works, books, book chapters, conclusion of course works, dissertations, theses, simple or complete abstracts published in proceedings of scientific events, and scientific review articles with data collected in more than 1 year, in order to avoid data overlap. Thus, we classified the selected final articles into three categories: (1) exclusive consumption of bushmeat; (2) consumption and trade bushmeat and (3) exclusive trade of bushmeat (Additional file 1). In our review we followed the guidelines and protocols PRISMA (Preferred Reporting Items for Systematic reviews and Meta-Analyses) [44].

**Fig. 1:**
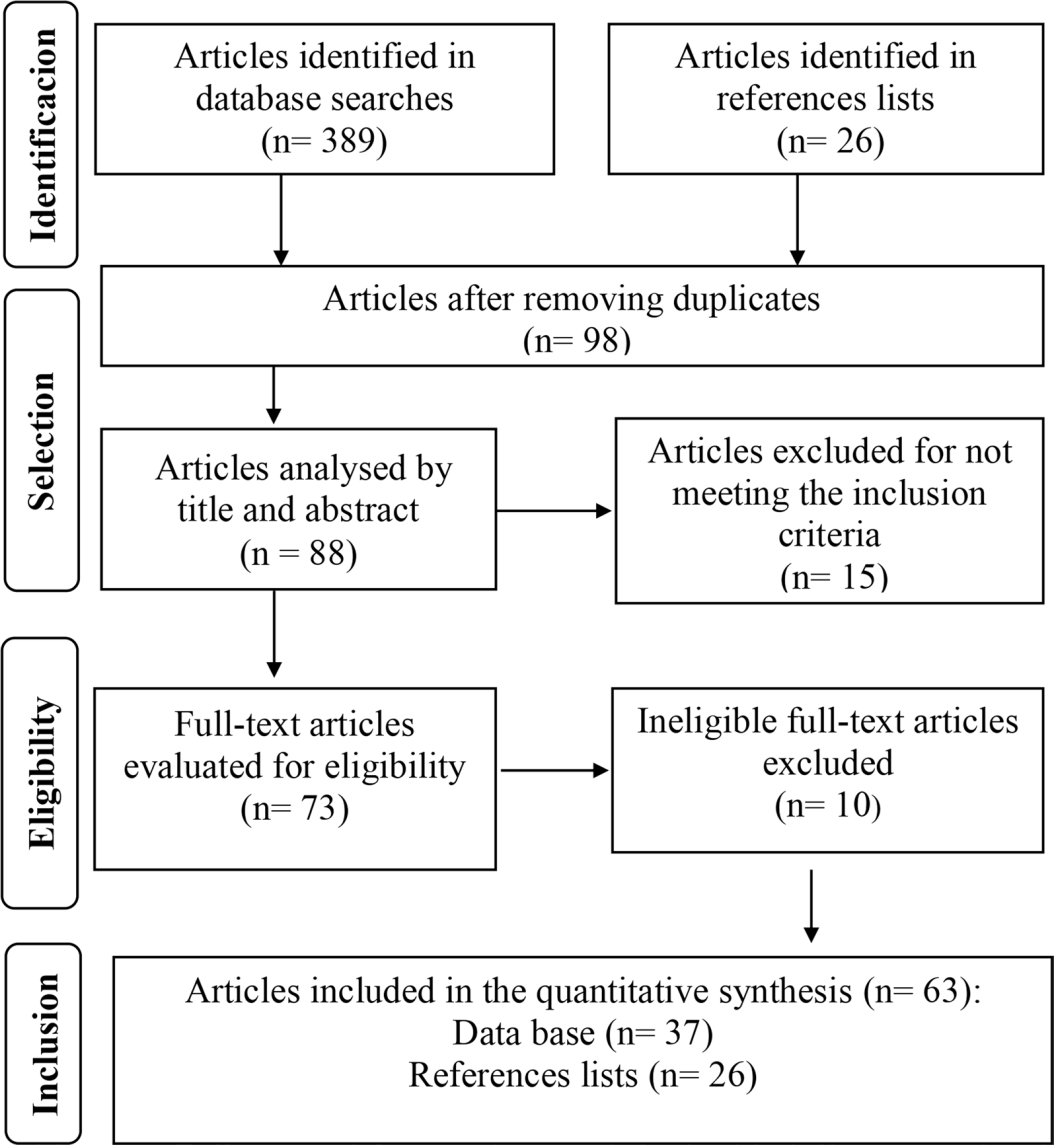
PRISMA flowchart with the stages of the process of identification and inclusion of articles. *Source*: Authors (2022)

In our database searches we identified a total of 7.557 results. In the primary searches we selected 389 articles: Google Scholar (*n* = 210), Portal de Periódicos da CAPES (*n* = 71), Science Direct (*n* = 14), Scopus (*n* = 34) and Web of Science (*n* = 60). From this total, we excluded 307 duplicate articles. We had 88 articles that had their title and abstract analyzed, and 15 were excluded for not meeting the inclusion criteria. We analyzed the full text of 73 articles, 10 of which were eliminated after applying the exclusion criteria. We additionally selected 26 articles identified from reference lists of articles in the databases. Thus, we included 63 articles in our final quantitative synthesis (Fig. 1).

### Data analysis

We used descriptive statistical analyses to compile the following information from the articles: species richness cited for bushmeat consumption and trade, nature or category of the papers, author(s), place and year of publication, type of environment or collection area, phytogeographic domain, drivers of consumption and trade, aspects of the trade chain: places of purchase and sale, forms of commercialization and actors involved, and aspects of wildlife conservation.

We applied a nonparametric Spearman's correlation analysis, after testing the assumptions of normality, to verify whether the richness of wild vertebrate species cited for consumption and trade in bushmeat was associated with the number of studies cited by states of the federation. All statistical analyses were performed using the R *version software* 4.1.2 [45], at a significance level of 5.0% (*p* < 0.05). In parallel, we developed a map to better understand the distribution of the number of papers per federative unit and the richness of species cited for consumption in each state. To prepare the map we used the Geographic Information System tool (SIG) *software QGIS, version* 3.16.

### Nomenclature and conservation status of wildlife

The systematic ordering and scientific nomenclature of taxa followed [46] for birds; [47] for mammals, [48] for reptiles and [49] for amphibians. We verified the conservation status of the species through the Red List of Threatened Species of the IUCN (International Union for Conservation of Nature) [50] and Official List of Brazilian Fauna Species Threatened with Extinction [51].

## Results

### Overview of research on consumption and trade of bushmeat in Brazil

**Fig. 2:**
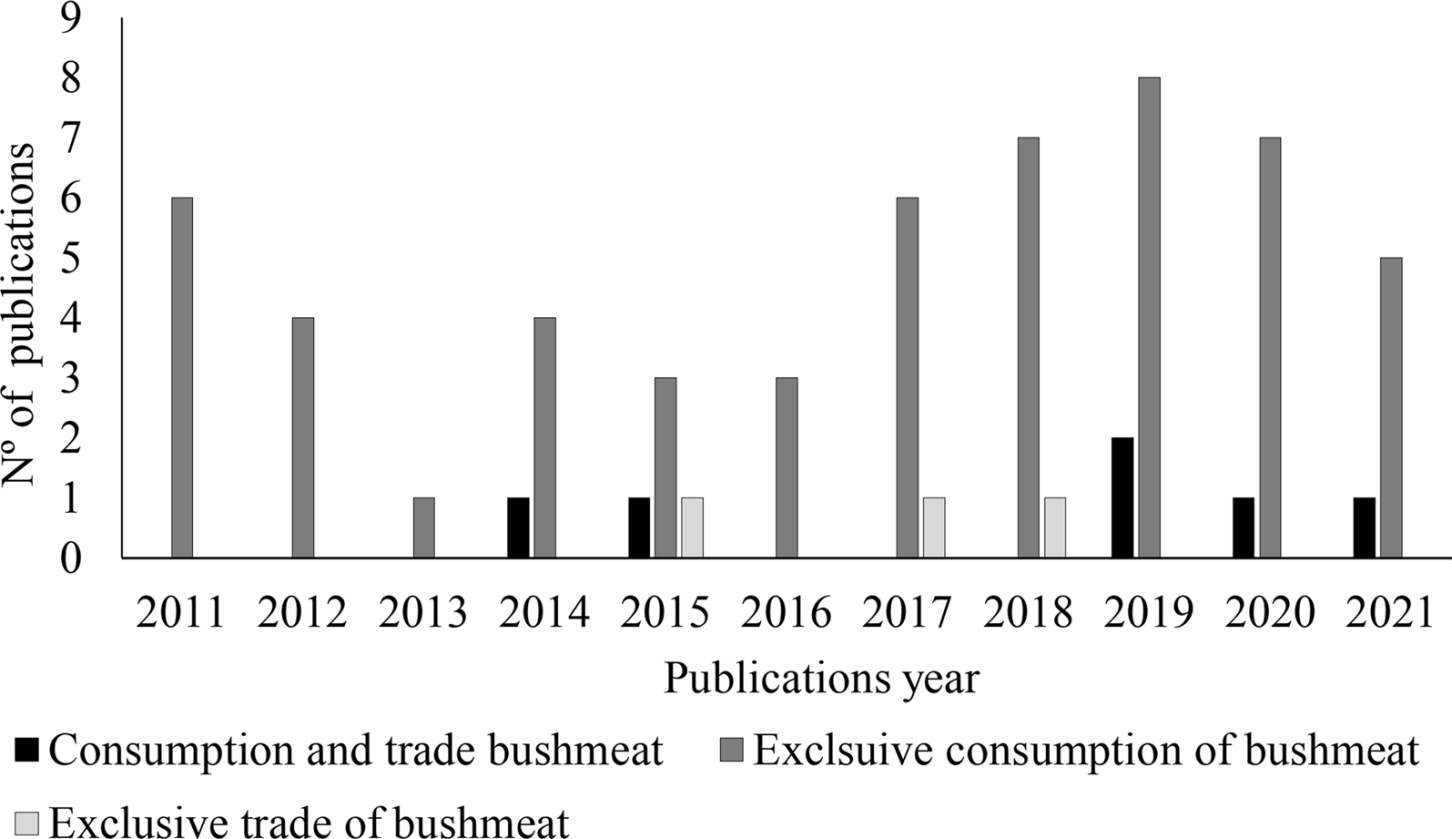
Temporal distribution of articles in the categories of consumption and trade of bushmeat in Brazil between 2011 and 2021. *Source*: Authors (2022)

The 63 scientific publications selected on the consumption and trade of wild vertebrate meat in the regions of Brazil were distributed in greater numbers in the bushmeat consumption category (*n* = 54; 85.7%), followed by bushmeat consumption and trade (*n* = 6; 9.5%) and exclusively bushmeat trade (*n* = 3; 4.7%) (Additional file 1). When analyzing the graph of the temporal distribution of the selected papers, we identified a considerable increase in the number of publications, especially in the bushmeat consumption category, in the last 5 years (2017–2021), while the other categories remained stable throughout years (Fig. 2).

Publications registered in 16 states of the federation were selected (i.e., 62% of the total). The majority in the states of the Northeast region of Brazil (*n* = 36; 57.1%), followed by the North (*n* = 26; 41.2%) and Southeast (*n* = 1; 1.6%). On the other hand, in the South or Central-West regions we did not have articles selected in our review.

In terms of phytogeographic domains, the papers were distributed across the following Brazilian biomes: Caatinga (*n* = 28; 44.4%), Amazonia (*n* = 26; 41.3%), Atlantic Forest (*n* = 3; 4.7%), Cerrado (*n* = 1; 1.6%) and two or more biomes (*n* = 5; 7.9%). Most articles had data collected in rural areas (*n* = 40; 63.4%), urban/rural (*n* = 8; 12.6%), urban/peri-urban (*n* = 5; 7.9%) and unspecified environments (*n* = 10; 15.8%).

### Richness of wild vertebrates for consumption for bushmeat

We recorded 321 wild vertebrates species in the 54 papers in the bushmeat consumption category. In this category, birds had the highest species richness (*n* = 170; 52.9%), followed by mammals (*n* = 107; 33.3%), reptiles (*n* = 40; 12.4%) and amphibians (*n* = 4; 1.2%) (Additional file 2).

Comparing the richness of birds species mentioned for consumption between regions of the country, we had a higher proportion of species recorded in states in the Northeast region of Brazil (*n* = 132; 77.2% of the total), followed by the North (*n* = 50; 29.2%) and Southeast (*n* = 2; 1.2%). We also had a higher proportion of birds families registered for consumption in the Northeast region of the country (*n* = 40 families; 86.9% of the total), followed by the North (*n* = 13; 28.3%) and Southeast (*n* = 2; 4.3%) (Additional file 2).

In general, the most representative families of birds cited for consumption in terms of species were Columbidae (*n* = 19), Cracidae (*n* = 18) and Tinamidae (*n* = 17). Among the birds species most commonly recorded in Brazil, especially in the northeastern states, we highlight the columbiformes *Zenaida auriculata* (avoante), *Columbina picui* (turtledove), *Columbina minuta* (cinnamon-winged turtledove), *Columbina talpacoti* (purple turtledove), *Leptotila verreauxi* (juriti-pupu) and *Patagioenas picazuro* (white-winged dove). Among the tinamiformes most cited in the papers were *Crypturellus parvirostris* (inhambu-chororó), *Crypturellus tataupa* (inhambu-chintã), *Nothura boraquira* (northeast quail) and *Nothura maculosa* (yellow quail). and among the cracids, the species *Pauxi tuberosa (*Razor-billed Curassow*)*, *Penelope superciliaris* (Rusty-margined Guan) and *Penelope jacquacu* (Spix's Guan) (Additional file 2).

Comparing the richness of mammal species cited for consumption between regions of the country, we had a higher proportion of species recorded in states in the North region (*n* = 77; 72.0% of the total), followed by the Northeast (*n* = 59; 55.1%) and Southeast (*n* = 9; 8.4%) (Additional file 2). Similar to species richness, we had a higher proportion of mammal families recorded in the states of the North region of the country (*n* = 24; 85.7%), followed by the Northeast (*n* = 21; 75%) and Southeast (*n* = 7; 25.0%).

In general, among the families with the greatest richness of species mentioned, we highlight Cebidae (*n* = 13), Atelidae (*n* = 11) and Dasyproctidae (*n* = 8). Among the species with the most records for consumption in the regions of the country, especially the North and Northeast we highlight *Pecari tajacu* (Collared Peccary), *Dasypus novemcinctus* (Nine-banded Armadillo), *Cuniculus paca* (Paca), *Euphractus sexcinctus* (Six-banded Armadillo), *Tamandua tetradactyla* (Tamandua Gray), *Hydrochoerus hydrochaeris* (capybara), *Tayassu pecari* (Peckerel), *Kerodon rupestris* (Rock cavy) and *Tapirus terrestris* (Tapir) (Additional file 2).

Comparing the richness of reptile species mentioned for consumption between regions of the country, we had a higher proportion of species mentioned in the states of the Northeast region (*n* = 28; 70.0% of the total), followed by the North (*n* = 17; 42.5%) and Southeast (*n* = 1; 2.5%). Similar to wealth, we recorded a higher proportion of families cited for consumption in the Northeast region (*n* = 14; 93.3%), followed by the North (*n* = 4; 26.7%) and Southeast (*n* = 1; 6.7%).

Among the reptile families with the highest species richness mentioned were Alligatoridae (*n* = 6), Podocnemididae (*n* = 5) and Cheloniidae (*n* = 4). Among the species of reptiles most commonly recorded in articles in the regions of the country, we highlight *Salvator merianae* (tegu), *Iguana iguana* (iguana), *Podocnemis unifilis* (yellow-headed sideneck turtle), *Podocnemis expansa* (Amazonian tortoise), *Podocnemis sextuberculata* (six-tubercled amazon river turtle), *Chelonoidis denticulatus* (yellow-footed tortoises) and *Caiman crocodilus* (common caiman) (Additional file 2). In the group of amphibians we had only 4 species mentioned for consumption, distributed in the families Leptodactylidae, Ranidae and Bufonidae, all with records in studies from the Northeast region of the country (Additional file 2).

**Fig. 3:**
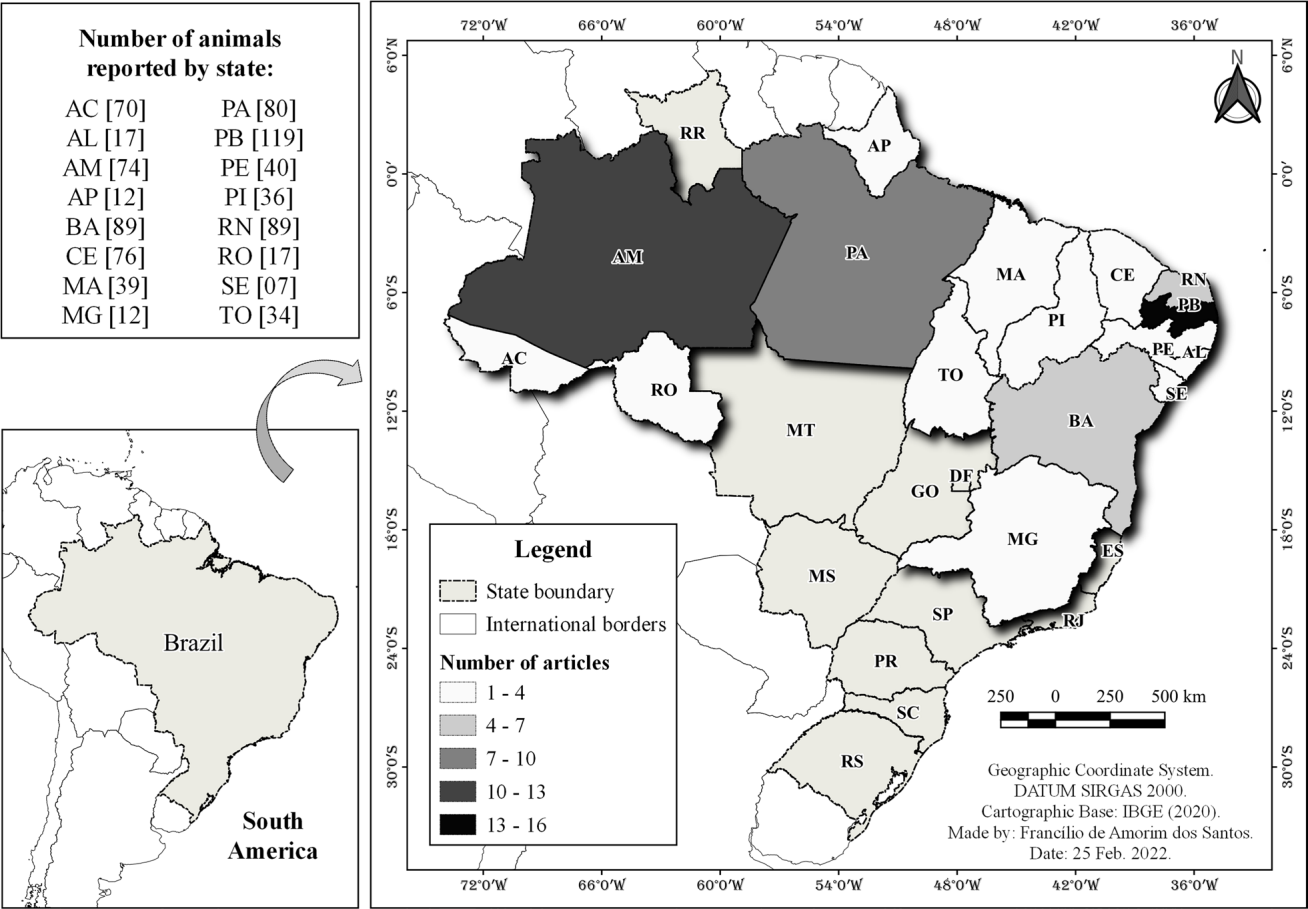
Distribution map of articles and number of wild vertebrate species cited for bushmeat consumption in Brazilian states in the period from 2011 to 2021. Legends: Brazilian states (*AC* Acre, *AL* Alagoas, *AP* Amapá, *AM* Amazonas, *BA* Bahia, *CE* Ceará, *ES* Espírito Santo, *GO* Goiás, *MA* Maranhão, *MG* Minas Gerais, *MS* Mato Grosso do Sul, *MT* Mato Grosso, *PA* Pará, *PE* Pernambuco, *PI* Piauí, *PB* Paraíba, *PR* Paraná, *RJ* Rio de Janeiro, *RR* Rondônia, *SC* Santa Catarina, *SE* Sergipe, *SP* São Paulo, *TO* Tocantins, *RN* Rio Grande do Norte, *RO* Roraima, *RS* Rio Grande do Sul) and federal district (*DF* Distrito Federal)

Our hypothesis that the richness of species cited for meat consumption would be positively associated with the amount of work carried out by states of the federation was confirmed (Spearman's *R* = 0.92; *p* < 0.05). The state of Paraíba had the highest richness of cited species (*n* = 119 spp.) and consequently more publications selected (*n* = 16 articles). On the other hand, the state of Sergipe had the lowest representation of species (*n* = 7 spp.) and consequently the lowest number of articles (*n* = 1) (Fig. 3).

### Richness of wild vertebrates in bushmeat trade works

We identified a total of 57 species of wild vertebrates mentioned in articles involving the bushmeat trade categories. The group of mammals presented the greatest richness of species mentioned (*n* = 29 spp.; 50.8%), followed by birds (*n* = 20; 35.1%) and reptiles (*n* = 8; 14.0%). Of the total recorded, we had only 8 species cited exclusively for the bushmeat trade (Additional file 3).

Comparing the number of birds species cited for the bushmeat trade between the regions of the country, we found a higher proportion of records in the Northeast region (*n* = 11; 55.0%), followed by the North (*n* = 10; 50.0%); we had no records in other regions of the country. In relation to families cited for commerce, the North region had the highest proportion of registrations (*n* = 4; 80.0%), followed by the Northeast (*n* = 3; 60.0%). Among the most representative birds families in species for trade in the country's regions, we highlight Columbidae (*n* = 8), Cracidae (*n* = 7) and Tinamidae (*n* = 3). Among the species with the highest frequency of records in the regions, we highlight *Crax sp.*, *Pauxi tuberosa*, *Nothura boraquira* (White-bellied Nothura) and *Crypturellus sp.* (Additional file 3).

The region of the country with the highest proportion of mammal species richness cited for trade was the North (*n* = 19; 65.5% of the total), followed by the Northeast (*n* = 12; 41.4%). In relation to families cited for commerce, the North region had the highest proportion of registrations (*n* = 13; 92.8%), followed by the Northeast (*n* = 8; 57.1%). Among the families cited for commercialization, we highlight Dasyproctidae (*n* = 5), Dasypodidade (*n* = 4) and Cervidae (*n* = 3) as the most significant species. Among the most frequent species in the articles we highlight *Cuniculus paca*, *Pecari tajacu*, *Tayassu pecari*, Dasypus sp., *Tapirus terrestris*, *Hydrochoerus hydrochaeris* and *Subulo gouazoubira* (brocket deer).

Among reptiles, we identified 8 species cited for the bushmeat trade, with a higher proportion of records in the North region of the country (*n* = 8; 100.0%) and Northeast region (*n* = 1; 12.5%). Similar to species richness, the highest proportion of family records was in the North region (*n* = 3; 100.0%) and Northeast region (*n* = 2; 66.7%). The families recorded were Podocnemididae (*n* = 4 spp.), Alligatoridae (*n* = 3) and Testudinidae (*n* = 1). The species *Caiman crocodilus*, *Chelonoidis denticulatus*, *Podocnemis unifilis* and *Podocnemis expansa* were among the most cited in the articles, especially from the northern region of the country. In the selected articles we did not record any amphibian species mentioned for the bushmeat trade.

### Drivers of bushmeat consumption and trade

Of the total articles selected, only 10 mentioned driving factors for the consumption and trade of bushmeat. The majority are in the bushmeat consumption category (*n* = 8) and the others (*n* = 2) in the bushmeat consumption and trade category. These articles were mainly concentrated in the North (*n* = 9; 90.0%) and Northeast (*n* = 1; 10.0%) regions of the country. We grouped the consumption and trade drivers cited into 6 categories: socioeconomic factors (*n* = 7 articles), biological (*n* = 4), environmental (*n* = 3), sociocultural (*n* = 3), spatial (*n* = 2) and demographic factors (*n* = 1). In Table 2 we summarize the main types of drivers of hunting meat consumption and trade registered by categories.

**Table 2:** Description of the factors drivers bushmeat consumption and trade identified in articles published in Brazil between 2011 and 2021

Drivers of bushmeat consumption and trade	Description of factors associated with the consumption and trade of bushmeat	References
Socioeconomic factors	Income, wealth, origin of head of household, level of education, socioeconomic indices (percentage of rural population, HDI, GDP per capita), number of hunters in the family, length of stay in the community, origin of bushmeat, family size, average family age, age of hunter, number of children, poverty probability index (PPI), number of years the head of household has left the rural area	[24, 26, 45, 52–55]
Biological factors	Biomass and species abundance	[24, 45, 52, 55, 56]
Sociocultural factors	Meat flavor preference, social structures: kinship relationships, social marketing: discount coupons, information campaign and engagement to reduce the consumption of wild bushmeat	[45, 50, 52, 55, 56]
Environmental factors	Seasonal variations: dry and rainy season, forest cover, landscape contexts	[54, 56, 57]
Spatial factors	Distance and remoteness from urban center	[54, 57]
Demographic factors	Location, river type, market access: frequency of city trips and boat traffic in communities	[53]

### Aspects of the bushmeat trade chain

We obtained information on aspects of the bushmeat trade chain from 8 of the 9 selected trade papers. In general, we recorded that the trade chain is dynamic and ramified with the presence of several authors, including specialized and diversified hunters, intermediaries, market sellers, restaurant owners and final customers (Table 3).

**Table 3:** Description of the aspects of the wild vertebrate meat trade chain listed in the bushmeat trade papers published

Aspects of the trade chain	Description of the main aspects of the trade chain	References
Main actors and drivers in the trade chain	Specialized and diversified hunters, middlemen/resellers, market traders and vendors, stallholders, street vendors, chopbars/restaurant owners, wholesalers, retailers and consumers from local fairs	[24, 28, 29, 34, 45–48]
Ways of selling bushmeat	Fresh (*in natura*), frozen, smoked, salted and live animal	[28, 34, 46–48]
Places to buy and sell bushmeat	Markets, street markets, formal and informal restaurants, chopbars, street food stalls, end customer homes, residence of hunters and intermediaries, rural communities, riverboats, ports, family homes	[24, 28, 29, 34, 45–48]
Main end customers and consumers in the trade chain	Urban residents (teachers, merchants, civil servants, wholesalers, family members, friends and neighbors); customers of chopbars and restaurants; settler or mestizo families; indigenous and non-indigenous consumers; tourists and shoppers	[24, 28, 29, 34, 45–48]

We identified that negotiations for the purchase and sale of bushmeat can occur directly, involving only hunters, traders, stallholders and end customers, or indirectly through intermediaries who carry out commercial transactions with end customers. Commercial transactions with end customers took place in the hunters own homes, and intermediaries were facilitated by the use of means of transport (e.g., motorcycles) and communication (e.g., cell phones) (Table 3).

In 5 five articles did we mention the ways in which bushmeat was sold. The meat was sold fresh (*in natura*), frozen, salted, smoked or even the animal alive. The main locations for buying and selling wild meat are most mentioned in our study in public markets, book fairs, chopbars, restaurants and residences of hunters, parents and intermediaries. We found that meat was intended mainly for urban residents, such as public officials, such as attackists, customers of small chopbars and restaurants, settler or mestizo families, indigenous and non-indigenous consumers and even tourists (Table 3).

### Conservation aspects of wild fauna

Of the 321 specific species in our study, the majority (*n* = 212; 77.3%) were included in the Least Concern (LC) category in the Red List of threatened species of the IUCN. Only 41 species were included in the endangered categories: Vulnerable (VU) (*n* = 29), Endangered (EN) (*n* = 10) and Critically Endangered (CR) (*n* = 2) (Additional file 2 and Additional file 3).

In the Endangered (EN) category we register the species of birds: *Crax blumenbachii* (red-billed curassow) and *Crax globulosa* (Wattled curassow). Among the mammals we have the species: *Sylvilagus brasiliensis* (Brazilian rabbit), *Ateles chamek* (Peruvian spider monkey), *Lagothrix poeppigii* (Silvery woolly monkey), *Leontopithecus chrysomelas* (Golden-headed lion tamarin), *Chiropotes satanas* (Black cuxiú) and *Sotalia fluviatilis* (Gray river dolphin) and 2 reptile species: *Chelonia mydas* (Green turtle) and *Lepidochelys olivacea* (Olive ridley turtle). In the category Critically Endangered (CR) we had the species: *Sapajus xanthosternos* (Yellow-breasted monkey) and *Eretmochelys imbricata* (Hawksbill turtle). In List of Brazilian Fauna Species Threatened, we identified 28 species included in threat categories: Vulnerable (VU; *n* = 21), Endangered (EN; *n* = 5) and Critically Endangered (CR; *n* = 2) (Additional file 2 and Additional file 3).

## Discussion

### Publications on bushmeat consumption and trade in Brazil

The growing in articles on bushmeat consumption and trade observed in recent years in our search is not surprising. Considering that in recent decades we have seen the insertion of new graduate programs in the country, the entry of new researchers, an increase in the number of newspapers that will certainly increase the Ethnozoological publications in the Brazil [41, 56, 58, 59]. Also, the increase of researchers with specific training in the fields of Ethnobiology and Ethnozoology and the existence of links between Brazilian researchers and from other international countries make Brazil a reference in this field in the Latin American context [56, 58–60].

As we verified, the North and Northeast regions of the country concentrated the largest number of articles selected in our research. The high number of publications in states in the Brazilian Northeast, such as Paraíba, Pernambuco and Bahia, for example, may be associated with research groups in Ethnobiology and related areas that have already consolidated themselves in different networks of scientific collaborations in these regions and in other Brazilian regions with international institutions and research centers [41, 58, 61].

The growth of scientific production in the North of the country may have been favored by the establishment of research centers in the areas of Environmental Sciences and Ecology, for example, such as the Museu Paraense Emílio Goeldi (MPEG) and Instituto Nacional de Pesquisas da Amazônia (INPA) [58]. In these centers experienced researchers have carried out, for example, several investigations into hunting monitoring activities and uses of wildlife by populations in the Amazon regions [22, 37, 39, 43, 62–64].

On the other hand, the low frequency of articles selected in the Southeast, Central-West and South regions of the country is possibly due to the scarcity of studies on hunting carried out in these regions, as pointed out by [41, 58] in publication review research Ethnozoological and hunting in Brazil, respectively. Thus, we confirm the need for more ethnozoological research in regions of the country that are still little explored. These investigations can provide more accurate information about species hunted for different uses by local communities and contribute to providing important data to be used in management strategies and wildlife conservation policies.

### Uses of wildlife for bushmeat consumption

In our research, the group of birds presented the greatest richness of species and families cited for consumption, with emphasis on records in states in the Northeast region of Brazil. These results reflect the trend of birds richness in the Brazilian semi-arid region (Caatinga biome), which is the largest compared to other groups of wild terrestrial vertebrates, with around 548 birds species recorded [65] against 156 mammal species [66] and 224 from reptiles [67].

The preference of populations in the Brazilian semi-arid region for the consumption of small animals with hunting potential, such as birds, may be related to the population decline of medium and large mammals species, such as deer, peccaries, pacas, agoutis, which have been suffering in recent years with the defaunation process [31, 66, 68]. Other aspects related to the greater wealth of birds cited for consumption are due to the way in which species can be captured, both through active hunting (e.g., using shotguns) and through the use of various non-selective hunting techniques [31, 69].

The preference for consuming birds from the families Columbidae, Tinamidae and Cracidae verified in our research reflects the importance of these groups, as they provide sources of proteins essential for the survival of rural and urban populations in Brazilian regions, especially in the semi-Brazilian region (Caatinga biome) [3, 4, 33, 70, 71]. In addition to protein value, aspects for example, meat flavor, abundance and availability, sociocultural contexts, ease of capture, gregarious behavior of small species (e.g., *Zenaida auriculata*), have been strong determinants for the exploitation of birds species by urban and rural populations in Brazil [34, 52, 72–74].

In our research, the group of mammals also stood out in the richness of species and families cited for consumption of bushmeat, with the majority of citations in the states of the northern region of the country. In the border regions of the Brazilian Amazon, for example, mammals represent the preferred species for consumption compared to reptiles and birds [53, 75–77]. These results also reflect the tropics scenario, in which mammals are the main hunting targets, supposedly because they provide greater protein return (body biomass) and meat supply [54, 57, 76, 78].

The preference for mammal species is not limited to body biomass alone; other aspects have been highlighted in the literature, such as the taste of the meat, abundance and availability of the species, ease of capture, cost–benefit and commercial value [7, 79–84]. For example, the species *Cuniculus paca* is one of the most appreciated for consumption in tropical regions, mainly due to the greater biomass and the flavor of the meat [35, 36, 81, 84, 85]. In South America, armadillos (*Dasypus* sp.), for example, are among the most hunted mammals for food or commercial consumption and their hunting is favored by a widespread perception that meat or products of animal origin are tastier or cleaner than those derived from household products animals [7, 34, 35, 80, 82, 86].

The consumption of reptile species was also highlighted in our research, with a greater incidence in the North region of the country. Previous studies have reported several species of chelonians, especially tortoises (*Chelonoidis denticulatus* and *Chelonoidis carbonaria*) and river tartars (*Podocnemis unifilis*, *Podocnemis sextuberculata* and *Podocnemis expansa*) being the most commonly hunted for consumption and trade in rural and urban regions of the Brazilian Amazon [35, 64, 87, 88]. According to [89], freshwater chelonians have been used as a food resource in the Amazon since the pre-Columbian period. Several historical records made by naturalists and colonizers attest to a significant exploitation of adult individuals and eggs of twelve-water chelonians in the Amazon, with emphasis on the genus *Podocnemis* [90, 91].

Currently, the consumption and trade of chelonian species to meet the demands of regional and even international markets is already significant. A recent study by [64] estimates that approximately 1.7 million turtles and tortoises can be consumed annually in urban centers in the central Amazon. Another recent study by [92] identified that turtle species, especially *Podocnemis unifilis* and *Podocnemis sextuberculata*, were among the main species whose meat and eggs were consumed and sold by hunters in tropical areas of the Eastern Amazon. In this context, we highlight the need to reinforce protection, inspection and awareness measures among local populations, in order to guarantee the sustainable management of Amazonian chelonian species, without putting them at risk of extinction.

The hunting importance of the *Salvator merianae* and *Iguana iguana* species highlighted in our study has also been widely recorded in different regions of the world, especially in Latin American countries [4, 93–95]. In addition to meat, other animal body parts (e.g., fat) have been used in traditional medicine to treat various diseases and illnesses, as evidenced by previous neotropical studies [42, 55, 96–98].

The low number of amphibian species for consumption found in our research may be associated with factors such as availability of other sources of animal protein, lack of eating habits, disease transmission, aversion and fear of the toxicity of these small animals in local populations [99, 100]. In this sense, these sets of factors make amphibians less attractive as a food source for local populations. Furthermore, in the literature there are few studies on the use of amphibians for consumption in the Neotropical regions of the world, including Brazil [101–103].

Therefore, our results reflect that in the current context, hunting and the consumption of game meat in Brazil are still common activities and play an important socioeconomic role. As we have shown, many species of wild vertebrates continue to provide a crucial source of protein for several rural and urban families, especially in the North and Northeast regions of the country, which do not have other sources of domestic protein.

### Wildlife uses in the bushmeat trade

Most of the scientific production on the use of wild fauna in the wild meat trade has been concentrated in the Northern region of Brazil, mainly because urban wild meat markets are already more established in Amazonian cities on the triple border (Brazil–Peru–Colombia), with a large volume of wild animals being sold [12, 35, 103, 104]. For example, there are significant urban wild meat markets in cities such as Pompéia, Ecuador [105], Abaetetuba in Pará, Brazil [106] and the cities of Letícia, Tabatinga, Benjamin Constant and Caballococha in the Amazon triple border region [12].

Since the trade of bushmeat is common in many Amazonian markets, it is difficult to obtain more information on the commerce of wild animal meat, mainly because the purchase and sale of wild animals is carried out illegally [107]. Furthermore, most of the information on bushmeat sold in South American cities derives largely from confiscations by environmental agencies [108] and, therefore, the quality of these data can be questioned in terms of its representativeness global trade.

The greater exploitation of mammal species in the bushmeat trade has also been recorded in other Neotropical studies [13, 109–112]. Our results showed a greater record of mammal species traded mainly in regions of the Brazilian Amazon. A study by [22], for example, identified the species *Tayassu pecari* (peccary) and *Tapirus terrestris* (tapir), together with chelonians (*Podocnemis unifilis* and *Podcnemis sextuberculata*) as responsible for 71.8% of the amount of bushmeat consumed in urban markets in the Central Amazon. Another recent study by [92] found that the meat of the species *Hydrochoerus hydrochaeris* (capybara) was the most widely cited among the species traded by local communities living in floodplain areas in the Brazilian Amazon.

Compared to mammals, the diversity of birds species exploited for the bushmeat trade in our study was lower. Although birds make up the smallest proportion of meat sold in markets, many species, including columbiformes, tinamiformes and cracids, are still hunted and traded as shown in neotropical literature [109–111, 113]. In the Brazilian Amazon basin, for example, a large volume of cracids (e.g., *Crax sp.*; *Penelope sp.*) have been illegally traded in urban and rural markets [12, 22, 37, 87, 90].

The reptile group had a greater participation of species cited for the bushmeat trade in the Northern region of Brazil. These results reflect the fact that in regions of the Brazilian Amazon, for example, several species of tortoises and turtles to the families Podocnemididae (e.g., *Podocnemis unifilis*, *Podocnemis expansa*) and Testudinidae (e.g., *Chelonoidis denticulatus*) are frequently consumed and valued as a local cultural delicacy [22, 35, 54, 114]. A recent study by [92], for example, identified 6 species of turtles of the genus Podocnemis as responsible for around 71% of the species cited for bushmeat trade in lowland communities in the eastern Amazon.

In the current context of increasing human populations, the bushmeat trade has increased dramatically over the last three decades in the tropics [13, 22, 112]. This increase in the illegal trade of wild animals for meat may have reached unsustainable levels, as the natural regenerative capacity of wildlife populations may not be high enough to meet the demand for bushmeat [112]. Against this background, unsustainable harvesting of wild meat in many tropical forests continues to threaten the survival of a wide range of wild species, as well as the food security of populations that depend on these resources as a means of survival [23, 26].

### Drivers of bushmeat consumption and trade

We identify a wide range of socioeconomic, biological, environmental and sociocultural factors associated with bushmeat consumption and trade. Interactions between humans and wildlife are affected by complex factors including income source, taste preference, culture, lack of alternative meat, meat price and wealth that regulate the ways in which local populations utilize wildlife resources [18, 38, 115–117].

However, hunting factors and bushmeat consumption are complex and can vary between different socio-ecological contexts and depending on usage patterns by local populations. For example, proximity to urban centers and local forest cover can affect the demand and supply of bushmeat. According to [35] found a relationship between remoteness from urban centers and a decrease in the availability of domestic sources of protein, resulting in high prices and a high demand for bushmeat.

Bushmeat consumption is also influenced by meat flavor preferences, as well as health, cultural and spiritual reasons [18, 115, 118]. Neotropical studies have demonstrated that cultural associations drive the consumption of game meat, as pointed out in the study by [38], which highlighted a strong association of beliefs (*taboos*), attitudes and social norms in understanding the consumption and preference of bushmeat among people urban inhabitants of the Brazilian Amazon. The study by [119], for example, in Bata, Equatorial Guinea highlighted ethnicity and nationality as the main key determinants of consumption. In this sense, understanding the sociocultural context and economic determinants of wildlife consumption and trade is critical to inform appropriate policy interventions to prevent overexploitation and promote the sustainable use of wildlife resources [57].

### Aspects of the bushmeat trade chain

Our results point to a dynamic bushmeat trade chain made up of several actors, similar to that described in studies carried out in countries in West Africa and Congo [120–122]. The existence of a diverse and dynamic trade chain highlights the complexity and extent of the problem of illegal wildlife trade. This chain involves different actors, from local hunters to intermediaries, international traffickers and end consumers, as shown in tropical studies [12, 37, 43, 87, 123].

In the current context, hunters represent true repositories of knowledge about fauna and the dynamics of exploitation of faunal, as they are directly involved in the capture and distribution of faunal products, in addition to evidently using animals for local uses [9, 124, 125]. Therefore, recognizing the importance of hunters' knowledge can be an important step toward the conservation and sustainable management of wildlife species.

The incorporation of new hunting technologies, including transportation (e.g., motorbikes, outboard boats) and communication (cell phones) by hunters, has facilitated wildlife trade transactions between different actors in the trade chain in the tropics [22, 27, 126]. On the other hand, the insertion of new technologies changed the patterns of consumption and trade of wild fauna in the tropics, increasing the demands for consumption and sale of wild animals, and consequently catalyzed contemporary processes of defaunation, with signs of reduction, extirpations and extinctions of faunal species [23, 43]. In this context, understanding the illegal wildlife trade chain and identifying the actors involved are key elements for developing effective conservation strategies to combat this problem on a global scale.

The diversification of ways of commercializing bushmeat observed in our research reflects the fact that wild animal meat has become an extremely versatile product, which can be obtained, transported, consumed immediately, stored, preserved for future consumption or even sold [52, 78, 80]. Salting and freezing are conservation methods widely used in many cultures around the world and in South American countries. The study carried out by [12], for example, found that bushmeat is commonly sold fresh in Colombia, smoked in Peru and salted or frozen in Brazil. In this context variations in the ways in which bushmeat is sold may reflect cultural differences, traditional food preparation and preservation practices, as well as regional consumption preferences.

Similar to what was found in regions of Central and West Africa, we highlight public markets and open-air markets as the main places for buying and selling bushmeat. In regions of Africa, for example, bushmeat markets are found in almost every city and play an important role as wildlife collection and trading centers [13, 109, 112].

In the current context, markets in the Brazilian Amazon basin also play an important role in the commercialization of wildlife products. In these markets, bushmeat is sold openly in open-air markets and can even be sold in the homes of hunters and intermediaries themselves, and there is, therefore, an intense commercial flow between Amazonian cities [22, 43, 62, 123, 127]. Therefore, understanding the patterns and dynamics of the bushmeat trade is an important step toward informing conservation policies, sustainable natural resource management and decision-making related to wildlife conservation and sustainable development of tropical regions and neotropics.

### Implications and challenges for wildlife conservation

Although most of the species recorded in our research were listed in non-endangered categories on international and national lists, there are several aspects that need to be considered regarding faunal conservation. For example, although a species may be classified as non-threatened on global and national lists, it may be locally threatened due to factors such as loss of habitat in specific areas, illegal hunting, use of wild meat by local populations, climate change and /or other human impacts [25, 26, 128, 129].

In our research, we listed two species of cracids in categories of threat of extinction at an international level. Special attention has been given to cracid populations, one of the most threatened birds families in the Americas [130, 131]. The loss of forest habitat and excessive hunting are considered key factors in the decline in populations of some cracid species such as the Wattled Mutum (*Crax globulosa*), White-browed Guan (*Penelope jacucaca*) and other galliformes [130–132].

In our research, Primates species also stood out in the number of species listed in endangered categories. A recently carried out study found that 68% of the world's Primates species for which data are available are listed in some category of threat of extinction and 93% are in population decline [133]. Most populations of Primates species are declining and threatened with extinction worldwide due to anthropogenic pressures resulting in habitat deforestation and fragmentation, increased urbanization, hunting for meat and other by-products [133–135].

Therefore, our results reflect an urgent need to implement conservation policies for populations of threatened species, such as primates, ungulates, marsupials and large birds (e.g., cracids) that have been excessively exploited to provide meat or other animal products in the tropics [23, 26, 34, 71, 73]. Furthermore, wildlife conservation requires an integrated approach to the various aspects involved in hunting activities, whether biological, political, economic, ecological or sociocultural. It is also essential to understand the dynamics and use relationships between local communities and wildlife in order to establish effective conservation strategies adapted to local needs and realities.

## Conclusions

Although the keywords used to search for publications on the consumption and trade of game meat in Brazil may produce biases and limitations to the generalization of conclusions, we consider that the articles reviewed may be a representative sample of the current situation of publications on the topic under study.

Our review study showed significant advances in publications on the consumption and trade of bushmeat in Brazil in recent years, with the majority of them concentrated in the North and Northeast regions of the country. We highlight the need for more research in regions that are still little explored, such as the South, Southeast and Central-West. Such investigation could provide greater information on the richness of target species for consumption and trade, directing more effective conservation strategies for target species.

In our research, we identified the group of birds and mammals as the most representative in terms of proportion of species richness and families cited for both consumption and trade of bushmeat in the regions of Brazil. In our research, we identified the group of birds and mammals as the most representative in terms of proportion of species richness and families cited for both consumption and trade of bushmeat in the regions of Brazil. These results reinforce the importance of game species from these groups, which are widely distributed and used for different uses by populations in urban and rural areas of the country, especially in the Brazilian semi-arid regions (Caatinga biome).

Our results also highlighted the need for more understanding on the part of research on the factors that drive the consumption and trade of bushmeat in different regions of the country, since few selected studies made mention of these factors. We also identified that the game meat trade chain is still poorly understood in Brazil, with detailed information on this trade only in the northern region of the country.

It is hoped that the information contained in this research can serve as a basis for future research and projects involving interactions between local communities and wild animals. We reinforce the urgent need for conservation measures and wildlife management strategies that have been continuously explored in hunting activities in different regions of the world.

### Supplementary Information

**Additional file 1.** List of articles on bushmeat consumption and trade selected in our study by category in our systematic review.

**Additional file 2.** List of wild vertebrate species cited in works on bushmeat consumption by regions in Brazil.

**Additional file 3.** List of wild vertebrates species cited in articles on consumption and trade bushmeat and exclusive trade bushmeat.

## Data Availability

The datasets used and/or analyzed during the current study are available from the supplementary material.

## Abbreviations

CAPES: Coordenação de Aperfeiçoamento de Pessoal de Nível Superior
IBGE: Instituto Brasileiro de Geografia e Estatística
INPA: Instituto Nacional de Pesquisas da Amazônia
IUCN: International Union for Conservation of Nature
MPEG: Museu Paraense Emílio Goeldi
MMA: Ministério do Meio Ambiente
PRISMA: Preferred Reporting Items for Systematic reviews and Meta-Analyses
SIG: Sistema de Informação Geográfica
