# Supplementary material for: Use of wild vertebrates for consumption and bushmeat trade in Brazil: a review
Source: J Ethnobiol Ethnomed. 2023 Dec 19;19:64. doi: 10.1186/s13002-023-00628-x (PMC10729539; doi:10.1186/s13002-023-00628-x)
Supplement: Supplementary file 1 — Additional file 1. List of articles on bushmeat consumption and trade selected in our study by category in our systematic review. [file 13002_2023_628_MOESM1_ESM.docx]

**Additional file 1-** List of articles on bushmeat consumption and trade selected in our study by category in our systematic review.

| Order | Authors | Tittle | Year of publication | Journal | Category of Article | |
| --- | --- | --- | --- | --- | --- | --- |
| 1. | Souto WMS, Lima RN, Sousa BFCF | Illegal bushmeat hunting and trade dynamics in a major road-hub region of the Brazilian Mid North | 2019 | Indian Journal of Traditional Knowledge | II |  |
| 2. | Barboza RRD, Lopes SF, Souto WMS, Fernandes-Ferreira H, Alves RRN | The role of game mammals as bushmeat in the Caatinga, northeast Brazil | 2016 | Ecology and Society | I |  |
| 3. | Mendonça LE, Vasconcellos A, Souto CM, Oliveira TP, Alves RRN | Bushmeat consumption and its implications for wildlife conservation in the semi-arid region of Brazil | 2016 | Regional Environmental Change | I |  |
| 4. | Da Silva JS, Nascimento ALB, Alves RRN, Albuquerque UP | Use of game fauna by Fulni-ô people in Northeastern Brazil: implications for conservation | 2020 | Journal of ethnobiology and ethnomedicine | I |  |
| 5. | Oliveira WSLD, Luna MDSO, Souto WMS, Alves RRN | Interactions between people and game mammals in a Brazilian semi-arid area | 2017 | Indian Journal of Traditional Knowledge | I |  |
| 6. | van Vliet N, Cruz D, Quiceno-Mesa MP, De Aquino LJN, Moreno J, Ribeiro R, Fa J | Ride, shoot, and call: wildlife use among contemporary urban hunters in Três Fronteiras, Brazilian Amazon. | 2015 | Ecology and Society | II |  |
| 7. | Torres PC, Morsello C, Parry L, Pardini R | Forest cover and social relations are more important than economic factors in driving hunting and bushmeat consumption in post-frontier Amazonia | 2021 | Biological Conservation | I |  |
| 8. | van Vliet N, Quiceno-Mesa MP, Cruz-Antia D, De Aquino, LJN, Moreno J, Nasi R | The uncovered volumes of bushmeat commercialized in the Amazonian trifrontier between Colombia, Peru & Brazil | 2014 | Ethnobiology Conservation | II |  |
| 9. | El Bizri HR, Morcatty TQ, Valsecchi J, Mayor P, Ribeiro JE, Vasconcelos Neto CF, Fa JE | Urban wild meat consumption and trade in central Amazonia | 2020 | Conservation Biology | II |  |
| 10. | El Bizri HR, Morcatty TQ, Ferreira JC, Mayor P, Neto CFV, Valsecchi J, Fa JE | Social and biological correlates of wild meat consumption and trade by rural communities in the Jutaí River basin, central Amazonia | 2020 | Journal of Ethnobiology | II |  |
| 11. | Torres PC, Morsello C, Parry L, Barlow J, Ferreira J, Gardner T, Pardini R et al. | Landscape correlates of bushmeat consumption and hunting in a post-frontier Amazonian region | 2018 | Environmental Conservation | I |  |
| 12. | Chaves WA, Valle DR, Monroe MC, Wilkie DS, Sieving KE, Sadowsky, B | Changing wildmeat consumption: an experiment in the cen-tral Amazon, Brazil | 2018 | Conservation Letters | I |  |
| 13. | Chaves WA, Wilkie DS, Monroe MC, Sieving KE | Market access and wild meat consump-tion in the central Amazon, Brazil | 2017 | Biological Conservation | I |  |
| 14. | Parry L, Barlow J, Pereira H | Wildlife harvest and consumption in Amazonia's urbanized wilderness | 2014 | Conservation Letters | I |  |
| 15. | Nunes AV, Guariento RG, Santos BA, Fischer E | Wild meat sharing amongnon-indigenous people in the southwestern Amazon. Behavioral | 2019 | Ecology and Sociobiology | I |  |
| 16. | Chaves WA, Valle D, Tavares AS, Morcatty TQ, Wilcove DS | Impacts of rural to urban mi-gration, urbanization, and generational changeon consumption of wild animals in the Amazon | 2021 | Conservation Biology | I |  |
| 17. | Mesquita GP, Rodríguez‐Teijeiro JD, Barreto LN | Patterns of mammal subsistence hunting in eastern Amazon, Brazil | 2018 | Wildlife Society Bulletin | I | |
| 18. | Teixeira JVS, dos Santos JS, Guanaes DHA, da Rocha WD, Schiavetti A | Wild Animals Used as Food Source in the Region of the Serra do Conduru State Park–PESC, Bahia, Brazil | 2020 | Research Square | I | |
| 19. | Melo RS, da Silva OC, Souto A, Alves RRN, Schiel N | The role of mammals in local communities living in conservation areas in the Northeast of Brazil: an ethnozoological approach. | 2014 | Tropical Conservation Science | I | |
| 20. | Castilho LC, De Vleeschouwer KM, Milner-Gulland, EJ, Schiavetti A | Hunting of mammal species in protected areas of the southern Bahian Atlantic Forest, Brazil | 2019 | Oryx | I | |
| 21. | Santos SS, Lucena RFP, Soares HKL, Soares VMS, Sales NS, Mendonça LET | Use of mammals in a semi-arid region of Brazil: an approach to the use value and data analysis for conservation. | 2019 | Journal of Ethnobiology and Ethnomedicine | I | |
| 22. | Lima JRB, Rebouças PLO, Santos CAB | Hunting and use of wildlife species in the semi-arid region of Brazil | 2020 | Amazonia Investiga | I | |
| 23. | Da Silva C, Ruiz-Esparza J, Azevedo CS, Ribeiro AS | Hunting and Trade of Columbidae in Northeast Brazil. | 2021 | Human Ecology | II | |
| 24. | Chaves LS, Alves RRN, Albuquerque UP | Hunters’ preferences and perceptions as hunting predictors in a semiarid ecosystem | 2020 | Science of The Total Environment | I | |
| 25. | Fernandes­Ferreira H, Mendonça SV, Albano C, Ferreira FS, Alves RRN | Hunting use and conservation of birds in Northeast Brazil | 2012 | Biodiversity and Conservation | I | |
| 26. | Santos SDS, Soares HKDL, Soares VMDS, Lucena, RFPD | Traditional knowledge and use of mammals in a rural community in the Sertaneja Depression (Paraíba State, Northeast Brazil) | 2019 | Indian Journal of Traditional Knowledge | I | |
| 27. | Bezerra DMMSQ, Araujo HFP, Alves RRN | The Use of Wild Birds by Rural Communities in the Semi-arid Region of Rio Grande do Norte State, Brazil | 2011 | Bioremediation, Biodiversity and Bioavailability | I | |
| 28. | Borges AKM, Ribeiro BDP, Alves RRN | Hunting, capture and wildlife use by communities in a semi-arid region of Northeastern Brazil | 2021 | Human Dimensions of Wildlife | I | |
| 29. | Santos CAB | Hunting Practices Among the Indigenous “Truká” in the Semiarid Region of Brazil | 2020 | Amazonia Investiga | I | |
| 30. | Ramos CGS, Santos RB, Santos RWC, Oliveira MA | Hunting in a community of waste pickers of recyclable materials in Rondônia, Brazil | 2020 | Revista Brasileira de Ciências da Amazônia | I | |
| 31. | Campos FL, Neto EMDFL, Costa-Neto EM, Ferreira F | Rural Populations of Alagoinhas and Herpetofauna: Knowledge, Uses and Interactions | 2021 | Research Square | I | |
| 32. | Cajaiba RL, Da Silva WB, Piovesan PRR | Animais silvestres utilizados como recurso alimentar em assentamentos rurais no município de Uruará, Pará, Brasil | 2015 | Desenvolvimento e Meio Ambiente | I | |
| 33. | Nunes AV, Vilela JS, Saldo PA, Santos BA, Fischer E | Conhecimento e uso de primatas por uma população extrativista no Vale do Juruá, Amazônia | 2017 | Biodiversidade Brasileira | I | |
| 34. | Reis YS, Valsecchi J, Queiroz H | Caracterização do uso da fauna silvestre para subsistência em uma unidade de conservação no oeste do Pará | 2018 | Biodiversidade Brasileira | I | |
| 35. | Damaceno AB, Ortega GP, Turci LCB | Uso da caça de subsistência no assentamento Santa Luzia, Cruzeiro do Sul, Acre | 2018 | Pubvet | I | |
| 36. | Souza JB, Alves RRN | Hunting and Wildlife use in an Atlantic Forest Remnant of Northeastern Brazil | 2014 | Tropical Conservation Science | I | |
| 37. | Dantas-Aguiar PR, Barreto RM, Santos-Fita D, Santos EB | Hunting activities and wild fauna use: a profile of queixo d’antas community, Campo Formoso, Bahia, Brazil | 2011 | Bioremediation, Biodiversity and Bioavailability | I | |
| 38. | Lemos LP, El Bizri HR, Valsecchi J, Santos AS, Koga DM, Silva FE | Caça de vertebrados no Parque Nacional da Serra do Divisor, Acre | 2018 | Biodiversidade Brasileira | I | |
| 39. | Loss ATG, Neto EMC, Flores FM | Aves silvestres utilizadas como recurso trófico pelos moradores do povoado de Pedra Branca, Santa Teresinha, Bahia, Brasil | 2014 | Gaia Scientia | I | |
| 40. | Alves RRN, Gonçalves MBR, Vieira WLC | Caca, uso e conservação de vertebrados no semiárido brasileiro | 2012 | Tropical Conservation Science | I | |
| 41. | Santos SL, Alves RRN, Mendonça LET | Fauna silvestre utilizada em comunidades rurais no semiárido paraibano | 2018 | Biodiversidade Brasileira | I | |
| 42. | Barbosa, EDO, Silva MDGB, Medeiros RO, Chaves MF | Atividades cinegéticas direcionadas à avifauna em áreas rurais do Município de Jaçanã, Rio Grande do Norte, Brasil | 2014 | Biotemas | I | |
| 43. | Lima JRF, Santos SS, Lucena RFP | Uso de recursos faunísticos em uma comunidade rural do semiárido da Paraíba-Brasil | 2018 | Revista Etnobiologia | I | |
| 44. | Figueiredo RAAD, Barros FB | Caçar, preparar e comer o ‘bicho do mato’: práticas alimentares entre os quilombolas na Reserva Extrativista Ipaú-Anilzinho (Pará) | 2016 | Boletim do Museu Paraense Emílio Goeldi. Ciências Humanas | I | |
| 45. | Nobrega VA, Barbosa JAA, Alves RRN | Utilização de aves silvestres por moradores do município de Fagundes, Semiárido paraibano: uma abordagem etno-ornitológica | 2012 | Sitientibus série Ciências Biológicas | I | |
| 46. | Santos SS | Conhecimento tradicional e utilização da fauna silvestre em São José da Lagoa Tapada, Paraíba, Brasil | 2019 | Revista Etnobiologia | I | |
| 47. | Bezerra DMM, de Araujo HFP, Alves RRN | Avifauna silvestre como recurso alimentar em áreas de semiárido no estado do Rio Grande do Norte, Brasil | 2011 | Sitientibus Série Ciências Biológicas | I | |
| 48. | Ferreira DSS, Campos CEC, Araujo AS | Aspectos da atividade de caça no Assentamento Rural Nova Canaã, município de Porto Grande, estado do Amapá | 2012 | Biota Amazônia | I | |
| 49. | Figueiredo RAA, Barros F B | "A Comida que vem da mata": conhecimentos tradicionais e práticas culturais de caçadores na Reserva Extrativista Ipaú-Anilzinho” | 2015 | Revista Fragmentos de Cultura-Revista Interdisciplinar de Ciências Humanas | I | |
| 50. | Barbosa, JJA, Aguiar, JO | Conhecimentos e usos da fauna por caçadores no semiárido brasileiro: um estudo de caso no estado da Paraíba, Nordeste do Brasil | 2015 | Biotemas | I | |
| 51. | Lima, RJPD, Barbosa EDO, Chaves M | Atividades de caça no semiárido Potiguar sob a perspectiva de estudantes | 2018 | Ambiente & Sociedade | I | |
| 52. | Lopes GP, Valsecchi J, Vieira TM, Amaral PV, Costa EWM | Hunting and hunters in lowland communities in the region of the middle Solimões, Amazonas, Brazil | 2012 | Scientific Magazine UAKARI | I | |
| 53. | Bezerra DMM, Araujo HFP, Alves RRN | Understanding the use of wild birds in a priority conservation area of Caatinga, a Brazilian tropical dry forest | 2020 | Environment, Development and Sustainability | I | |
| 54. | Guimarães C, Palha M, Tourinho M | Estratégias e dinâmica de caça na ilha de Colares, Pará, Amazônia Oriental | 2019 | Biota Amazônia | I | |
| 55. | Soares VMS, Soares LHK, Lucena PRF, Barbosa RR | Conhecimento, uso alimentar e conservação da avifauna cinegética: estudo de caso no município de Patos, Paraíba, Brasil | 2018 | Interciencia | I | |
| 56. | Souza LS, Progênio M, Souza LS, Araújo Santos, FG | Consumption of wild animals in extractive communities in the State of Acre, Brazilian Amazon | 2021 | Biota Amazônia | I | |
| 57. | Campos FL, Neto EMDFL, Costa-Neto EM, Ferreira F | Rural Populations of Alagoinhas and Herpetofauna: Knowledge, Uses and Interactions | 2021 | Research Square | I | |
| 58. | Paula M, Xerente V, Pezzuti J | Hunting and monitoring: community-based research in Xerente Indigenous Land, Brazilian Cerrado | 2017 | Human Ecology Review | I | |
| 59. | Barbosa JAA, Nobrega VA, Alves RRN | Hunting practices in the semiarid region of Brazil | 2011 | Indian Journal of Traditional Knowledge | I | |
| 60. | Pinto LCL, Mateus MB, Pires MRS | Conhecimentos e usos da fauna terrestre por moradores rurais da Serra do Ouro Branco, Minas Gerais, Brasil | 2012 | Interciencia | I | |
| 61. | Santos, M. K. P., Ruiz-Miranda, C. R., & Sampaio, D. T. | Comércio de Caça na Região da Estação Ecológica Raso da Catarina, Bahia, Brasil | 2018 | Biodiversidade Brasileira | III | |
| 62. | van Vliet N, Quiceno MP, Cruz D, De Aquino LJN, Yagüe B, Schor T, Hernandez S, Nasi R, et al. | Bushmeat networks link the forest to urban areas in the trifrontier region between Brazil, Colombia, and Peru | 2015 | Ecology & Society | III | |
| 63. | Quaresma AÂN, Da Silva KJM, Silva PMP | Aspectos da comercialização da carne de animais silvestres na feira municipal de Abaetetuba - Pará | 2017 | Revista Científica Multidisciplinar Núcleo do Conhecimento | III | |

Legends: (I) exclusive consumption bushmeat; (II) consumption and trade bushmeat e (III) exclusive trade bushmeat.
