# Supplementary material for: Use of wild vertebrates for consumption and bushmeat trade in Brazil: a review
Source: J Ethnobiol Ethnomed. 2023 Dec 19;19:64. doi: 10.1186/s13002-023-00628-x (PMC10729539; doi:10.1186/s13002-023-00628-x)
Supplement: Supplementary file 2 — Additional file 2. List of wild vertebrate species cited in works on bushmeat consumption by regions in Brazil. [file 13002_2023_628_MOESM2_ESM.docx]

**Additional file 2 –** List of wild vertebrate species cited in works on bushmeat consumption by regions in Brazil.

| Táxon/Species | | | Brazilian regions cited in articles | | | List References* | Status Conservation | |
| --- | --- | --- | --- | --- | --- | --- | --- | --- |
|  |  |  | NE | N | S |  | IUCN (2021) | MMA  (2022) |
| AVES |  | |  |  |  |  |  |  |
| Rheiformes |  | |  |  |  |  |  |  |
| Rheidae |  | |  |  |  |  |  |  |
| *Rhea americana* (Linnaeus, 1758) | | | AL | TO |  | 22, 58 | NT | NL |
| Ciconiiformes |  | |  |  |  |  |  |  |
| Ciconiidae |  | |  |  |  |  |  |  |
| *Ciconia maguari* (Gmelin, 1789) | | |  | AM |  | 10 | LC | NL |
| *Ciconia sp.* | | |  | AC |  | 56 | - | - |
| *Jabiru mycteria* (Lichtenstein, 1819) | | |  | PA |  | 44 | LC | NL |
| Charadriiformes | | |  |  |  |  |  |  |
| Charadriidae |  | |  |  |  |  |  |  |
| *Vanellus chilensis* (Molina, 1782) | | | BA, PB |  |  | 3, 36, 39 | LC | NL |
| Jacanidae |  | |  |  |  |  |  |  |
| *Jacana jacana* (Linnaeus, 1766) | | | PE, PB |  |  | 4, 40, 46, 55 | LC | NL |
| Pelecaniformes | | |  |  |  |  |  |  |
| Ardeidae |  | |  |  |  |  |  |  |
| *Tigrisoma lineatum* (Boddaert, 1783) | | | PE, PB |  |  | 4, 40, 44, 46, 53 | LC | NL |
| *Butorides striata* (Linnaeus, 1758) | | | RN, PB |  |  | 40, 51 | LC | NL |
| *Ardea alba* (Linnaeus, 1758) | | | RN |  |  | 47 | LC | NL |
| *Egretta thula (*Molina, 1782) | | | PB |  |  | 55 | LC | NL |
| Coraciiformes | |  |  |  |  |  |  |  |
| Alcedinidae | |  |  |  |  |  |  |  |
| *Megaceryle torquata* (Linnaeus, 1766) | | | PB |  |  | 46 | LC | NL |
| Phalacrocoracidae | |  |  |  |  |  |  |  |
| *Nannopterum brasilianum* (Gmelin, 1789) | | | RN |  |  | 27 | LC | NL |
| Cariamiformes | |  |  |  |  |  |  |  |
| Cariamidae | |  |  |  |  |  |  |  |
| *Cariama cristata* (Linnaeus, 1766) | | | RN, PB, PE, BA, AL | PA, TO |  | 3, 4, 22, 27, 28, 29, 32, 41, 43, 46, 47, 51, 53, 55, 58 | LC | NL |
| Podicipediformes | |  |  |  |  |  |  |  |
| Podicipedidae | |  |  |  |  |  |  |  |
| *Podilymbus podiceps* (Linnaeus, 1758) | | | PE, BA |  |  | 29 | LC | NL |
| Caprimulgiformes | | |  |  |  |  |  |  |
| Caprimulgidae | |  |  |  |  |  |  |  |
| *Nyctidromus albicollis* (Gmelin, 1789) | | | PB |  |  | 40 | LC | NL |
| *Nyctiphrynus ocellatus* (Tschudi, 1844) | | | RN |  |  | 42, 51 | LC | NL |
| *Nyctidromus hirundinaceus* (Spix, 1825) | | | PB |  |  | 45 | LC | NL |
| Gruiformes | |  |  |  |  |  |  |  |
| Psophiidae | |  |  |  |  |  |  |  |
| *Psophia leucoptera* (Spix, 1825) | | |  | AM, AC |  | 8, 15, 38, 56 | NT | NL |
| *Psophia viridis* (Spix, 1825) | | |  | PA |  | 44 | VU | VU |
| *Psophia sp.* | | |  | PA |  | 34 | - | - |
| Rallidae | |  |  |  |  |  |  |  |
| *Gallinula chloropus* (Linnaeus, 1758) | | | PB |  |  | 3, 36, 50, 59 | LC | NL |
| *Gallinula galeata* (Lichtenstein, 1818) | | | RN, PB, CE |  |  | 25, 27, 42, 45, 46, 47, 51, 53, 55 | LC | NL |
| *Porphyriops melanops* (Vieillot, 1819) | | | CE |  |  | 25 | LC | NL |
| *Porphyrio martinica* (Linnaeus, 1766) | | | PB |  |  | 25, 40, 46, 53, 55 | LC | NL |
| Aramidae | | |  |  |  |  |  |  |
| *Aramide*s *ypecaha* (Vieillot, 1819) | | | CE |  |  | 25 | LC | NL |
| *Aramides cajaneus* (Statius Muller, 1776) | | | PB, CE | PA |  | 25, 36, 40, 44, 53 | LC | NL |
| *Aramides mangle* (Spix, 1825) | | | CE |  |  | 25 | LC | NL |
| *Aramides saracura* (Spix, 1825) | | |  | AC |  | 56 | LC | NL |
| *Aramides sp.* | | |  | TO |  | 58 | - | - |
| *Pardirallus maculatus* (Boddaert, 1783) | | | CE |  |  | 25 | LC | NL |
| *Aramus guarauna* (Linnaeus, 1766) | | | PB, PE, BA |  |  | 29, 46, 53, 55 | LC | NL |
| Tinamiformes | |  |  |  |  |  |  |  |
| Tinamidae | |  |  |  |  |  |  |  |
| *Nothura maculosa* (Temminck, 1815) | | | RN, PB, PE, BA, CE |  |  | 3, 4, 25, 36, 39, 40, 41, 51, 53, 55 | LC | NL |
| *Nothura boraquira* (Spix, 1825) | | | RN, PB, PE, BA, CE |  |  | 3, 4, 25, 27, 28, 29, 39, 40, 41, 45, 46, 47, 51, 53, 55 | LC | NL |
| *Nothura sp.* | | | BA |  |  | 39 | - | - |
| *Crypturellus tataupa* (Temminck, 1815) | | | RN, PB, BA, CE |  |  | 3, 25, 36, 37, 39, 40, 41, 42, 45, 46, 50, 51, 53, 55, 59 | LC | NL |
| *Crypturellus sp.* | | | BA | AM, PA, AC | MG | 6, 8, 32, 39, 56, 60 | - | - |
| *Tinamus major* (Gmelin, 1789) | | |  | AM |  | 14, 15, 56 | LC | NL |
| *Tinamus tao* (Temminck, 1815) | | |  | AM, PA |  | 14, 15, 32, 56 | VU | VU |
| *Tinamus solitarius* (Vieillot, 1819) | | | BA |  |  | 18 | NT | NL |
| *Tinamus guttatus* (Pelzeln, 1863) | | |  | AM, PA, AC |  | 14, 32, 38, 44, 49, 56 | NT | NL |
| *Tinamus sp.* | | |  | AM, RO, PA |  | 6, 7, 9, 10, 12, 13, 30, 34 | - | - |
| *Crypturellus parvirostris* (Wagler, 1827) | | | RN, PB, PE, BA, CE | TO |  | 3, 4, 18, 25, 36, 39, 40, 41, 42, 45, 46, 50, 51, 53, 55, 58, 59 | LC | NL |
| *Crypturellus soui* (Hermann, 1783) | | | BA |  |  | 18 | LC | NL |
| *Crypturellus noctivagus* (Wied, 1820) | | | PB, BA |  |  | 39, 45 | NT | NL |
| *Rhynchotus rufescens* (Temminck, 1815*)* | | | PB, PE, BA |  |  | 18, 29, 39, 55 | LC | NL |
| *Crypturellus undulatus* (Temminck, 1815) | | |  | AC, TO |  | 56, 58 | LC | NL |
| *Crypturellus cinereus* (Gmelin, 1789) | | |  | AC |  | 56 | LC | NL |
| *Crypturellus strigulosus* (Temminck, 1815) | | |  | AC |  | 56 | LC | NL |
| Galliformes | |  |  |  |  |  |  |  |
| Cracidae | |  |  |  |  |  |  |  |
| *Mitu sp.* | | |  | AM, AC |  | 9, 56 | - | - |
| *Nothocrax urumutum* (Spix, 1825) | | |  | AM |  | 14 | LC | NL |
| *Crax sp.* | | |  | AM |  | 6, 8, 9 | - | - |
| *Crax blumenbachii* (Spix, 1825) | | | BA |  |  | 18 | EN | EN |
| *Crax globulosa* (Spix, 1825) | | |  | AM |  | 14, 52 | EN | EN |
| *Crax fasciolata* (Spix, 1825) | | |  | PA, TO |  | 32, 34, 49, 58 | VU | NL |
| *Crax fasciolata pinima* (Pelzeln, 1870) | | |  | PA |  | 44 | VU | CR |
| *Pauxi tuberosa* (Spix, 1825) | | |  | AM, RO, PA, AC |  | 10, 12, 13, 14, 30, 32, 34, 38, 44, 49, 52 | NT | NL |
| *Aburria cujubi* (Pelzeln, 1858) | | |  | AC |  | 14, 38, 56 | VU | VU |
| *Aburria sp.* | | |  | AM |  | 6, 8 | - | - |
| *Penelope sp.* | | |  | PA | MG | 11, 14, 32, 60 | - | - |
| *Penelope jacquacu* (Spix, 1825) | | | PB | AM, AC |  | 6, 8, 10, 12, 13, 15, 38, 46, 53, 56 | LC | NL |
| *Penelope superciliaris* (Temminck, 1815) | | | PI, PB, BA, CE | PA, TO |  | 1, 18, 25, 36, 39, 44, 45, 46, 53, 58 | NT | NL |
| *Penelope jacucaca* (Spix, 1825) | | | PB, CE |  |  | 25, 41, 53, 55 | VU | VU |
| *Penelope marail* (Statius Muller, 1776) | | |  | PA |  | 32 | LC | NL |
| *Penelope pileata* (Wagler, 1830) | | |  | PA |  | 34 | VU | VU |
| *Ortalis araucuan* (Spix, 1825) | | | BA |  |  | 18 | LC | NL |
| *Ortalis guttata* (Spix, 1825) | | | PB, BA | AC |  | 36, 38, 39, 56 | LC | NL |
| Odontophoridae | |  |  |  |  |  |  |  |
| *Odontophorus capueira* (Spix, 1825) | | | CE |  |  | 25 | LC | NL |
| Opisthocomiformes | |  |  |  |  |  |  |  |
| Opisthocomidae | |  |  |  |  |  |  |  |
| *Opisthocomus hoazin* (Statius Muller, 1776) | | |  | AC |  | 35, 56 | LC | NL |
| Suliformes | |  |  |  |  |  |  |  |
| Anhingidae | |  |  |  |  |  |  |  |
| *Anhinga anhinga* (Linnaeus, 1766 | | |  | AM |  | 9 | LC | NL |
| Anseriformes | |  |  |  |  |  |  |  |
| Anatidae | |  |  |  |  |  |  |  |
| *Cairina moschata* (Linnaeus, 1758) | | |  | AM, PA, AC |  | 9, 10, 12, 14, 32, 38, 44, 49, 52, 54 | LC | NL |
| *Cairina sp.* | | |  | AC |  | 56 | - | - |
| *Amazonetta brasiliensis* (Gmelin, 1789) | | | RN, PB, PE, BA, CE |  |  | 4, 25, 29, 45, 47, 53 | LC | NL |
| *Dendrocygna autumnalis* (Linnaeus, 1758) | | | PB |  |  | 14, 43 | LC | NL |
| *Netta erythrophthalma* (Wied, 1833) | | | PB, BA |  |  | 25, 55 | LC | NL |
| *Nomonyx dominicus* (Linnaeus, 1766) | | | RN, PB, CE |  |  | 25, 40, 51 | LC | NL |
| *Dendrocygna viduata* (Linnaeus, 1766) | | | RN, PB, PE, BA, CE |  |  | 25, 27, 29, 39, 40, 46, 47, 51, 53, 55 | LC | NL |
| *Sarkidiornis sylvicola* (Ihering & Ihering, 1907*)* | | | PB |  |  | 40, 55 | LC | NL |
| Anhimidae | | |  |  |  |  |  |  |
| *Anhima cornuta* (Linnaeus, 1766) | | | PI |  |  | 1 | LC | NL |
| Piciformes | |  |  |  |  |  |  |  |
| Ramphastidae | |  |  |  |  |  |  |  |
| *Ramphastos sp.* | | | BA | AM, RO, PA |  | 14, 15, 18, 30, 32, 34 | - | - |
| *Ramphastos tucanus* (Linnaeus, 1758) | | |  | AC |  | 56 | VU | NL |
| *Colaptes melanochloros* (Gmelin, 1788) | | | BA |  |  | 39 | LC | NL |
| Galbuliformes | |  |  |  |  |  |  |  |
| Bucconidae | | |  |  |  |  |  |  |
| *Nystalus maculatus* (Gmelin, 1788) | | | RN, PB, BA |  |  | 3, 39, 42, 51 | LC | NL |
| Galbulidae | |  |  |  |  |  |  |  |
| *Galbula ruficauda* (Cuvier, 1816) | | | CE |  |  | 25 | LC | NL |
| Cuculiformes | |  |  |  |  |  |  |  |
| Cuculidae | |  |  |  |  |  |  |  |
| *Guira guira* (Gmelin, 1788) | | | PB, PE, BA |  |  | 3, 4, 39 | LC | NL |
| *Coccyzus melacoryphus* (Vieillot, 1817) | | | RN, PB, CE |  |  | 3, 25, 40, 42, 51 | LC | NL |
| *Coccyzus euleri* (Cabanis, 187) | | | CE |  |  | 25 | LC | NL |
| *Piaya cayana* (Linnaeus, 1766) | | | PB, CE |  |  | 25, 28 | LC | NL |
| *Crotophaga major* (Gmelin, 1788) | | | CE |  |  | 25 | LC | NL |
| *Crotophaga ani* (Linnaeus, 1758) | | | RN, BA, CE |  |  | 25, 39, 51 | LC | NL |
| [Nyctibiiformes](https://www.wikiaves.com.br/wiki/nyctibiiformes) | |  |  |  |  |  |  |  |
| [Nyctibiidae](https://www.wikiaves.com.br/wiki/nyctibiidae) | |  |  |  |  |  |  |  |
| *Nyctibius* griseus (Gmelin, 1789) | | | PB |  |  | 45 | LC | NL |
| Passeriformes | |  |  |  |  |  |  |  |
| Turdidae | |  |  |  |  |  |  |  |
| *Turdus rufiventris* (Vieillot, 1818) | | | RN, PB, BA, CE |  |  | 3, 25, 39 ,40, 42, 51 | LC | NL |
| *Turdus leucomelas* (Vieillot, 1818) | | | PB, CE |  |  | 25, 46 | LC | NL |
| *Turdus amaurochalinus* (Cabanis, 1850) | | | CE |  |  | 25 | LC | NL |
| *Turdus sp.* | | | PB, BA, AL |  |  | 22, 39, 41 | - | - |
| Icteridae | |  |  |  |  |  |  |  |
| *Psarocolius decumanus* (Pallas, 1769) | | | BA |  |  | 18 | LC | NL |
| *Agelaioides fringillarius* (Spix, 1824) | | | PB |  |  | 40, 43 | LC | NL |
| *Gnorimopsar chopi* (Vieillot, 1819) | | | RN |  |  | 42 | LC | NL |
| *Molothrus bonariensis* (Gmelin, 1789) | | | RN |  |  | 42, 51 | LC | NL |
| *Icterus jamacaii* (Gmelin, 1788) | | | RN, PB |  |  | 43, 51 | LC | NL |
| *Icterus pyrrhopterus* (Vieillot, 1819) | | | PB |  |  | 46 | LC | NL |
| *Icterus cayanensis* (Linnaeus, 1766) | | | RN |  |  | 51 | LC | NL |
| Furnariidae | | |  |  |  |  |  |  |
| *Pseudoseisura cristata* (Spix, 1824) | | | AL |  |  | 22 | LC | NL |
| *Furnarius leucopus* (Swainson, 1838) | | | RN |  |  | 42, 51 | LC | NL |
| *Synallaxis frontalis* (Pelzeln, 1859) | | | RN |  |  | 42, 51 | LC | NL |
| Formicariidae | |  |  |  |  |  |  |  |
| *Chamaeza campanisona* (Lichtenstein, 1823) | | | CE |  |  | 25 | LC | NL |
| Cotingidae | | |  |  |  |  |  |  |
| *Procnias averano* (Hermann, 1783) | | | RN |  |  | 25 | LC | NL |
| Tyrannidae | | |  |  |  |  |  |  |
| *Pitangus sulphuratus* (Linnaeus, 1766) | | | CE |  |  | 39, 40, 51 | LC | NL |
| *Tyrannus melancholicus* (Vieillot, 1819) | | | RN |  |  | 42, 51 | LC | NL |
| *Empidonomus varius* (Vieillot, 1818) | | | RN |  |  | 42 | LC | NL |
| *Phyllomyias fasciatus* (Thunberg, 1822) | | | RN |  |  | 51 | LC | NL |
| Thraupidae | | |  |  |  |  |  |  |
| *Thraupis sayaca* (Linnaeus, 1766) | | | RN |  |  | 39, 42, 51 | LC | NL |
| *Coereba flaveola* (Linnaeus, 1758) | | | RN |  |  | 42, 51 | LC | NL |
| *Stilpnia cayana* (Linnaeus, 1766) | | | RN, PB |  |  | 42, 51 | LC | NL |
| *Paroaria dominicana* (Linnaeus, 1758) | | | RN |  |  | 42, 46, 51 | LC | NL |
| *Coryphospingus pileatus* (Wied, 1821) | | | RN, PB |  |  | 42, 51 | LC | NL |
| *Sporophila albogularis* (Spix, 1825) | | | RN |  |  | 42, 46, 51 | LC | NL |
| *Sporophila nigricollis* (Vieillot, 1823) | | | RN |  |  | 51 | LC | NL |
| *Volatinia jacarina* (Linnaeus, 1766) | | | CE |  |  | 42, 51 | LC | NL |
| *Sicalis flaveola* (Linnaeus, 1766) | | | RN, PB |  |  | 46, 51 | LC | NL |
| Thamnophilidae | | |  |  |  |  |  |  |
| *Taraba major* (Vieillot, 1816) | | | RN |  |  | 42 | LC | NL |
| *Myrmorchilus strigilatus* (Wied, 1831) | | | RN |  |  | 42 | LC | NL |
| Troglodytidae | | |  |  |  |  |  |  |
| *Troglodytes musculus* (Naumann, 1823) | | | RN |  |  | 42, 51 | - | NL |
| Mimidae | | |  |  |  |  |  |  |
| *Mimus saturninus* (Lichtenstein, 1823) | | | RN, PB |  |  | 42, 43, 51 | LC | NL |
| Passerellidae | | |  |  |  |  |  |  |
| *Zonotrichia capensis* (Statius Muller, 1776) | | | RN |  |  | 42, 51 | LC | NL |
| Fringillidae | | |  |  |  |  |  |  |
| *Euphonia chlorotica* (Linnaeus, 1766) | | | RN |  |  | 42 | LC | NL |
| Cardinalidae | | |  |  |  |  |  |  |
| *Cyanoloxia brissonii* (Lichtenstein, 1823) | | | RN, PB |  |  | 43, 51 | LC | NL |
| Corvidae | | |  |  |  |  |  |  |
| *Cyanocorax cyanopogon* (Wied, 1821) | | | RN, PB |  |  | 46, 51, 53 | LC | NL |
| Hirundinidae | | |  |  |  |  |  |  |
| *Progne tapera* (Linnaeus, 1766) | | | RN |  |  | 51 | LC | NL |
| Psittaciformes | |  |  |  |  |  |  |  |
| Psittacidae | |  |  |  |  |  |  |  |
| *Eupsittula cactorum* (Kuhl, 1820) | | | PB |  |  | 3, 46 | LC | NL |
| *Amazona sp.* | | |  | PA, AC, TO |  | 14, 32, 34, 56, 58 | - | - |
| *Amazona aestiva* (Linnaeus, 1758) | | | RN, PB |  |  | 43, 51 | NT | NL |
| *Ara sp.* | | |  | PA, AC |  | 32, 34, 56 | - | - |
| *Ara macao* (Linnaeus, 1758) | | |  | AC |  | 38 | LC | NL |
| *Ara chloropterus* (Gray, 1859 | | |  | PA |  | 44, 49 | LC | NL |
| *Ara ararauna* (Linnaeus, 1758) | | |  | TO |  | 58 | LC | NL |
| *Forpus xanthopterygius* (Spix, 1824) | | | RN |  |  | 42, 51 | LC | NL |
| *Aratinga sp.* | | |  | AC |  | 56 | - | - |
| Strigiformes | |  |  |  |  |  |  |  |
| Strigidae | |  |  |  |  |  |  |  |
| *Megascops choliba* (Vieillot, 1817) | | | BA |  |  | 39 | LC | NL |
| *Athene cunicularia* (Molina, 1782) | | | BA |  |  | 39 | LC | NL |
| *Glaucidium brasilianum* (Gmelin, 1788) | | | RN |  |  | 42, 51 | LC | NL |
| Apodiformes | |  |  |  |  |  |  |  |
| Trochilidae | |  |  |  |  |  |  |  |
| *Chlorostilbon lucidus* (Shaw, 1812) | | | PB |  |  | 46 | LC | NL |
| Columbiformes | |  |  |  |  |  |  |  |
| Columbidae | |  |  |  |  |  |  |  |
| *Leptotila verreauxi* (Bonaparte, 1855) | | | RN, PB, PE, BA, CE |  |  | 3,25,27,29,36,39,40,41,42,45,46,47,51,53,55 | LC | NL |
| *Leptotila rufaxilla* (Richard & Bernard, 1792) | | | PB, BA, CE |  |  | 25,28,37,43,50,59 | LC | NL |
| *Leptotila sp.* | | | SE | PA, AC |  | 14,23,32,56 | - | - |
| *Zenaida auriculata* (Des Murs, 1847) | | | RN, PB, PE, BA, CE, SE |  |  | 3,4,22,23,25,27,28,29,40,41,42,45,46,47,50,51,53,55,59 | LC | NL |
| *Patagioenas picazuro* (Temminck, 1813) | | | RN, PB, PE, BA, CE, SE |  |  | 3,4,23,25,27,29,39,40,41,42,46,47,51,53,55 | LC | NL |
| *Patagioenas cayennensis* (Bonnaterre, 1792) | | | PB, BA |  |  | 18,36 | LC | NL |
| *Patagioenas speciosa* (Gmelin, 1789) | | | PB |  |  | 36 | LC | NL |
| *Claravis pretiosa* (Ferrari-Perez, 1886) | | | RN, PB |  |  | 3,40,45,46,51,53,55 | LC | NL |
| *Columbina picui* (Temminck, 1813) | | | RN, PB, PE, BA, CE, SE |  |  | 3,22,23,25,27,28,29,39,40,41,42,45,46,47,51,53,55 | LC | NL |
| *Columbina minuta* (Linnaeus, 1766) | | | RN, PB, PE, CE, SE |  |  | 3,4,23,25,27,28,36,40,42,45,46,47,50,51,53,55,59 | LC | NL |
| *Columbina talpacoti* (Temminck, 1810) | | | RN, PB, BA, CE, SE | AC |  | 3,23,25,27,28,36,39,40,41,42,45,46,47,51,53,55,56 | LC | NL |
| *Columbina squammata* (Lesson, 1831) | | | RN, PB, BA, CE, SE |  |  | 23,25,27,39,40,45,46,47,51,53,55 | LC | NL |
| *Columbina passerina* (Linnaeus, 1758) | | | PB, CE |  |  | 25,43,45 | LC | NL |
| *Columbina sp.* | | | PB, BA | PA |  | 32,39,50 | - | - |
| *Claravis pretiosa* (Ferrari-Perez, 1886) | | | CE |  |  | 25 | LC | NL |
| *Columba palumbus* (Linnaeus, 1758) | | | BA |  |  | 37 | LC | NL |
| *Columba livia* (Gmelin, 1789) | | | PB |  |  | 55 | LC | NL |
| *Streptopelia sp.* | | | PB |  |  | 59 | - | - |
| *Streptopelia decaocto* (Frivaldszky, 1838) | | | PB |  |  | 59 | LC | NL |
| Accipitriformes | |  |  |  |  |  |  |  |
| Accipitridae | |  |  |  |  |  |  |  |
| *Elanus leucurus* (Vieillot, 1818) | | | RN, PB, PE |  |  | 4,41,42 | LC | NL |
| *Urubitinga urubitinga* (Gmelin, 1788) | | | RN, PB |  |  | 40,51 | LC | NL |
| *Geranoaetus melanoleucus* (Vieillot, 1819) | | | PB |  |  | 40,55 | LC | NL |
| *Buteogallus schistaceus* (Sundevall, 1850) | | | PB |  |  | 41 | LC | NL |
| *Harpagus bidentatus* (Latham, 1790) | | | PB |  |  | 41 | LC | NL |
| *Heterospizias meridionalis* (Latham, 1790) | | | PB |  |  | 46,55 | LC | NL |
| *Rupornis magnirostris* (Gmelin, 1788) | | | PB |  |  | 55 | LC | NL |
| Falconiformes | |  |  |  |  |  |  |  |
| Falconidae | |  |  |  |  |  |  |  |
| *Caracara plancus* (J. F. Miller, 1777) | | | PB, PE |  |  | 4,46 | LC | NL |
| *Herpetotheres cachinnans* (Linnaeus, 1758) | | | PB, BA |  |  | 39,53 | LC | NL |
| *Micrastur ruficollis* (Vieillot, 1817) | | | PB |  |  | 40 | LC | NL |
| MAMMALIA | |  |  |  |  |  |  |  |
| Artiodactyla | |  |  |  |  |  |  |  |
| Cervidae | |  |  |  |  |  |  |  |
| *Mazama sp*. | | |  | AM, PA, AC | MG | 9,10,12,13,14,20,34,35,51,54,56,60 | - | - |
| *Mazama americana* (Erxleben, 1777) | | | PI, RN, MA, BA | AM, RO, PA, AC, AP, TO |  | 2,6,7,8,11,14,15,17,18,30,32,36,44,48,49,58 | DD | NL |
| *Mazama nemorivaga* (Cuvier, 1817) | | | MA | RO, AC, PA |  | 7,14,15,17,30,32,38 | LC | NL |
| *Subulo gouazoubira* (Fischer, 1824) | | | PI, RN, PB, MA, PE, BA, CE | AM, AP, PA, TO |  | 1,2,6,8,11,17,18,19,24,26,29,37,40,46,48,58 | LC | NL |
| *Ozotoceros bezoarticus* (Linnaeus, 1758) | | |  | PA, TO |  | 44,49,58 | NT | VU |
| *Odocoileus virginianus* (Zimmermann, 1780) | | |  | PA |  | 44 | LC | NL |
| Tayassuidae | | |  |  |  |  |  |  |
| *Tayassu pecari* (Link, 1795) | | | MA, CE | AM, AC, PA, RO, AP, TO |  | 6,7,8,9,10,12,13,14,17,19,30,34,35,38,44,48,49,56,58 | VU | VU |
| *Pecari tajacu* (Linnaeus, 1758) | | | PI, PB, MA, PE, BA | AM, RO, PA, AC, AP, TO | MG | 1,6,7,8,9,10,11,12,13,14,15,17,18,20,26,29,30,32,34,35,37,38,44,46,48,49,54,56,58,60 | LC | NL |
| Cingulata | |  |  |  |  |  |  |  |
| Chlamyphoridae | |  |  |  |  |  |  |  |
| *Cabassous unicinctus* (Linnaeus, 1758) | | | PB, MA, CE | AM, AC, RO, PA, TO | MG | 11,17,19,20,30,34,36,38,44,49,56,58,60 | LC | NL |
| *Priodontes maximus* (Kerr, 1792) | | | MA | AM, AC, PA, AP |  | 8,17,38,42,44,48 | VU | VU |
| *Tolypeutes tricinctus* (Linnaeus, 1758) | | | BA, CE | PA, AP |  | 19,37,44,48 | VU | EN |
| *Cabassous tatouay* (Desmarest, 1804) | | | BA |  |  | 18 | LC | NL |
| *Euphractus sexcinctus* (Linnaeus 1758) | | | PI, RN, PB, MA, PE, BA, CE | PA, AP, TO |  | 1,2,3,4,5,17,18,19,21,22,24,26,28,29,32,36,37,40,43,44,46,47,49,50,51,58,59 | LC | NL |
| Dasypodidae | | |  |  |  |  |  |  |
| *Dasypus sp.* | | |  | AM, PA |  | 6, 8,9,14,15,34,54 | - | - |
| *Dasypus kappleri* (Krauss, 1862) | | | MA | AM, PA, AC |  | 14,17,38,44 | LC | NL |
| *Dasypus novemcinctus* (Linnaeus, 1758) | | | PI, RN, PB, MA, PE, BA, CE, | AM, AC, PA, RO, TO |  | 1,2,3,5,7,11,15,17,18,19,20,21,22,24,26,29,30,35,36,37,38,40,41,43,46,50,51,56,58,59 | LC | NL |
| *Dasypus septemcinctus* (Linnaeus, 1758) | | | PI, PB | TO |  | 1,14,36,58 | LC | NL |
| *Dasypus beniensis* (Lönnberg, 1942) | | |  | RO |  | 30 | - | NL |
| Rodentia | |  |  |  |  |  |  |  |
| Sciuridae | |  |  |  |  |  |  |  |
| *Hadrosciurus sp.* | | |  | AC, AM |  | 15,56 | - | - |
| *Hadrosciurus igniventris* (Wagner, 1842) | | |  | PA |  | 54 | - | NL |
| Echimyidae | | |  |  |  |  |  |  |
| *Thrichomys apereoides* (Lund, 1941) | | | PI, RN, PB, MA, PE, CE |  |  | 2,3,4,5,19,24,26,40,41,46 | LC | NL |
| *Thrichomys laurentius* (Thomas, 1904) | | | PB |  |  | 21 | DD | NL |
| Caviidae | | |  |  |  |  |  |  |
| *Kerodon rupestris* (Wied, 1820) | | | PI, RN, PB, MA, BA, PE, CE, AL |  |  | 2,3,4,19,21,22,24,26,28,29,40,41,43,46,50,51,59 | LC | VU |
| *Galea spixii* (Wagler, 1831) | | | PI, RN, PB, MA, BA, PE, CE, AL |  |  | 2,3,4,5,19,21,22,26,28,29,36,40,41,43,46,51 | LC | NL |
| *Cavia aperea* (Erxleben, 1777) | | | PI, RN, PB, MA, PE |  |  | 2,17,24,50,59 | LC | NL |
| *Hydrochoerus hydrochaeris* (Linnaeus, 1766) | | | PI, PB, MA, PE, BA, AL | AM, RO, PA, AC, AP, TO | MG | 1,2,5,7, 9,14, 17,18,22,29,30,32,34,36,38,44,48,49,52,54,56,58,60 | LC | NL |
| Erethizontidae | | |  |  |  |  |  |  |
| *Coendou bicolor* (Tschudi, 1844) | | |  | AM |  | 6 | LC | NL |
| *Coendou prehensilis* (Linnaeus, 1758) | | | PI, RN, PB, MA | AM, PA, AC, TO |  | 2,35,36,38,44,54,58 | LC | NL |
| *Coendou insidiosus* (Olfers, 1818) | | | BA |  |  | 18 | LC | NL |
| *Chaetomys subspinosus* (Olfers, 1818) | | | BA |  |  | 18,20 | VU | NL |
| Cuniculidae | | |  |  |  |  |  |  |
| *Cuniculus paca* (Linnaeus, 1758) | | | PI, PB, MA, BA | AM, RO, PA, AC, AP, TO | MG | 1,2,6,7,8,9,10,11,12,13,14,15,17,18,20,21,26,30,32,34,35,36,38,44,48,49,54,58,60 | LC | NL |
| Dasyproctidae | | |  |  |  |  |  |  |
| *Dasyprocta fuliginosa* (Wagler, 1832) | | | MA | AM, AC |  | 6,8,9,10,14,15,17,38 | LC | NL |
| *Dasyprocta aguti* (Linnaeus, 1766) | | | BA | PA |  | 17,35 | - | NL |
| *Dasyprocta leporina* (Linnaeus, 1758) | | |  | AM, AC |  | 7,11,18,20,37,44,49,54 | LC | NL |
| *Myoprocta pratti* (Pocock, 1913) | | |  | AC |  | 15,38 | LC | NL |
| *Myoprocta sp.* | | | MA | AM, AC |  | 56 | - | - |
| *Dasyprocta prymnolopha* (Wagler, 1831) | | | PI, RN, PB, MA, CE |  |  | 1,2,19,24,36 | LC | NL |
| *Dasyprocta azarae* (Lichtenstein, 1823) | | |  | AP |  | 48 | DD | NL |
| *Dasyprocta sp.* | | |  | RO, PA, TO |  | 30,32,34,58 | - | - |
| Cricetidae | |  |  |  |  |  |  |  |
| *Oligoryzomys sp.* | | | BA |  |  | 18 | - | - |
| *Nectomys squamipes* (Brants, 1827) | | | BA |  |  | 18 | LC | NL |
| Dinomyidae | |  |  |  |  |  |  |  |
| *Dinomys branickii* (Peters, 1873) | | |  | AC |  | 38 | LC | NL |
| Perissodactyla | |  |  |  |  |  |  |  |
| Tapiridae | |  |  |  |  |  |  |  |
| *Tapirus terrestris* (Linnaeus, 1758) | | |  | AM, RO, AC, PA, AP, TO |  | 6,8,9,10,11,12,13,14,15,30,32,34,38,44,48,56,58 | VU | VU |
| Pilosa | |  |  |  |  |  |  |  |
| Megalonychidae | |  |  |  |  |  |  |  |
| *Choloepus didactylus* (Linnaeus, 1758) | | | MA |  |  | 17 | LC | NL |
| *Choloepus hoffmanni* (Peters, 1858) | | |  | PA |  | 44 | LC | NL |
| Myrmecophagidae | |  |  |  |  |  |  |  |
| *Tamandua tetradactyla* (Linnaeus, 1758) | | | PI, RN, PB, MA, PE, BA, CE | PA, AC, TO | MG | 1,2,3,17,18,19,20,21,22,24,26,28,29,32,35,36,37,38,40,43,46,51,58,60 | LC | NL |
| *Myrmecophaga tridactyla* (Linnaeus, 1758) | | |  | PA, AP, TO |  | 32,34,44,48,51,58 | VU | VU |
| Bradypodidae | | |  |  |  |  |  |  |
| *Bradypus variegatus* (Schinz, 1825) | | | PB, BA | PA |  | 18,32,36,44,49 | LC | NL |
| *Bradypus torquatus* (Illiger, 1811) | | | BA |  |  | 18 | VU | VU |
| *Bradypus tridactylus* (Linnaeus, 1758) | | |  | PA |  | 44,49 | LC | NL |
| *Bradypus sp.* | | |  | PA |  | 54 | - | - |
| Carnivora | |  |  |  |  |  |  |  |
| Mephitidae | |  |  |  |  |  |  |  |
| *Conepatus semistriatus* (Boddaert, 1785) | | | PI, RN, PB, MA, PE, CE |  |  | 2,3,4,5,19,21,22,24,26,28,40,41,43,46,50,51,59 | LC | NL |
| Mustelidae | | |  |  |  |  |  |  |
| *Eira barbara* (Linnaeus, 1758) | | | PI, PB, MA, BA | AM, AC |  | 2,9,18,36,56 | LC | NL |
| *Galictis vittata* (Schreber, 1776) | | | PI, RN, PB, MA |  |  | 2,28 | LC | NL |
| *Galictis cuja* (Molina, 1782*)* | | | RN, PB |  |  | 21,26,51 | LC | NL |
| *Lontra longicaudis* (Olfers, 1818) | | | PI, PB, MA, BA, PA |  |  | 2, 18,36,44 | NT | NL |
| Procyonidae | |  |  |  |  |  |  |  |
| *Nasua nasua* (Linnaeus, 1766) | | | PI, PB, MA, BA | PA, AC, TO | MG | 2,14,15,17,18,20,34,36,38,44,54,56,58,62 | LC | NL |
| *Procyon cancrivorus* (Storr, 1780) | | | PI, RN, PB, MA, BA |  |  | 2,18,21,26,36,51,58 | LC | NL |
| *Potos flavus* (Schreber, 1774) | | | BA |  |  | 18,20 | LC | NL |
| Canidae | | |  |  |  |  |  |  |
| *Speothos venaticus* (Lund, 1839) | | | PI, PE |  |  | 2 | NT | VU |
| *Cerdocyon thous* (Linnaeus, 1766) | | | PI, RN, PB, MA, BA, CE | TO |  | 1,2,18,19,21,22,26,46,51,58 | LC | NL |
| *Chrysocyon brachyurus* (Illiger, 1815) | | | AL |  |  | 22 | NT | VU |
| *Lycalopex vetulus* (Lund, 1842) | | |  | TO |  | 58 | NT | NL |
| Felidae | | |  |  |  |  |  |  |
| *Panthera onca* (Linnaeus, 1758) | | | PI, PB, MA, CE | PA, RO | MG | 2,19,26,30,34,37,44,46,60 | NT | VU |
| *Puma concolor* (Linnaeus, 1771) | | | PI, PB, MA, CE | RO |  | 2,19,21,26,30,46 | LC | NL |
| *Herpailurus yagouaroundi* (É. Geoffroy Saint-Hilaire, 1803) | | | PI, RN, PB, MA | AM, TO |  | 2,5,15,21,26,28,40,46,51,58 | LC | VU |
| *Leopardus pardalis* (Linnaeus, 1758) | | | PI, RN, PB, MA |  |  | 2,5,21,26,46 | LC | NL |
| *Leopardus wiedii* (Schinz, 1821) | | | PI, RN, PB, MA, | PA |  | 2,34,54 | NT | VU |
| *Leopardus tigrinus* (Schreber, 1775) | | | PI, RN, PB, MA, PE, CE |  |  | 2,4,19,22,24,28,40,41 | VU | EN |
| *Leopardus sp.* | | | RN, PB, BA | TO |  | 18,21,26,46,51,58 | - | - |
| Didelphimorphia | |  |  |  |  |  |  |  |
| Didelphidae | |  |  |  |  |  |  |  |
| *Didelphis marsupialis* (Linnaeus, 1758) | | | PI, MA | PA, AC |  | 2,6,35,44 | LC | NL |
| *Didelphis albiventris* (Lund, 1840) | | | PI, RN, PB, MA, PE, BA, CE |  |  | 2,5,19,24,26,28,29,36,37,40,41,50,51,59 | LC | NL |
| *Didelphis aurita* (Wied-Neuwied, 1826) | | | BA |  |  | 12 | LC | NL |
| *Didelphis sp.* | | |  | PA |  | 32,54 | - | - |
| Lagomorpha | |  |  |  |  |  |  |  |
| Leporidae | |  |  |  |  |  |  |  |
| *Sylvilagus brasiliensis* (Linnaeus, 1758) | | | PI, PB, MA, BA |  | MG | 2,18,21,24,32,36,38,60 | EN | NL |
| Sirenia | |  |  |  |  |  |  |  |
| Trichechidae | |  |  |  |  |  |  |  |
| *Trichechus inunguis* (Natterer, 1883) | | |  | AM |  | 9,10,12,52 | VU | VU |
| Primates | |  |  |  |  |  |  |  |
| Atelidae | |  |  |  |  |  |  |  |
| *Alouatta nigerrima* (Lönnberg, 1941) | | |  | PA |  | 14,34 | LC | NL |
| *Alouatta juara* (Elliot, 1910) | | |  | AM, AC |  | 10,15,33,38,52 | LC | NL |
| *Alouatta seniculus* (Linnaeus, 1766) | | |  | AM |  | 9 | LC | NL |
| *Alouatta caraya* (Humboldt, 1812) | | |  | AC |  | 3 | NT | NL |
| *Alouatta belzebul* (Linnaeus, 1766) | | |  | PA |  | 44,49 | VU | VU |
| *Ateles chamek* (Humboldt, 1812) | | |  | AC, AM |  | 10,33,38,56 | EN | VU |
| *Alouatta sp.* | | |  | PA, AC |  | 32,56 | - | - |
| *Lagothrix cana* (É. Geoffroy St.-Hilaire,1812) | | |  | AM |  | 14 | VU | NL |
| *Lagothrix poeppigii* (Schinz, 1844) | | |  | AM, PA |  | 10,38 | EN | NL |
| *Lagothrix lagotricha* (Humboldt, 1812) | | |  | AM |  | 6,8 | VU | NL |
| Cebidae | | |  | AM, AC |  |  |  |  |
| *Cebus unicolor* (Spix, 1823) | | |  | AC |  | 15,33 | VU | NL |
| *Cebus albifrons* (Humboldt, 1811) | | |  | AM, AC |  | 38 | LC | NL |
| *Sapajus macrocephalus* (Spix, 1823) | | |  |  |  | 15, 33, 38 | LC | NL |
| *Sapajus xanthosternos* (Wied-Neuwied, 1820) | | | BA |  |  | 18, 20 | CR | EN |
| *Sapajus apella* (Linnaeus, 1758) | | |  | PA, AC, TO |  | 14, 34, 56, 58 | LC | NL |
| *Aotus nigriceps* (Dollman, 1909) | | |  | AM, AC |  | 15, 33, 38 | LC | NL |
| *Aotus azarae* (Humboldt, 1812) | | |  | PA |  | 44 | LC | NL |
| *Aotus sp.* | | |  | AC |  | 56 | - | - |
| *Callicebus melanochir* (Wied-Neuwied, 1820) | | | BA |  |  | 18, 20 | VU | NL |
| *Leontopithecus chrysomelas* (Kuhl, 1820) | | | BA |  |  | 20 | EN | NL |
| *Callithrix jacchus* (Linnaeus, 1758) | | | RN, PB |  |  | 21, 36, 51 | LC | NL |
| *Saimiri boliviensis* (I. Geoffroy St.-Hilaire & Blainville, 1834) | | |  | AC |  | 33, 56 | LC | NL |
| *Saimiri macrodon* (Elliot, 1907) | | |  | AC |  | 38 | - | NL |
| Pitheciidae | | |  |  |  |  |  |  |
| *Pithecia vanzolinii* (Hershkovitz, 1987) | | |  | AM, AC |  | 15, 33 | DD | NL |
| *Pithecia irrorata* (Gray, 1843) | | |  | PA |  | 34 | DD | NL |
| *Pithecia monachus* (É. Geoffroy St.-Hilaire, 1812) | | |  | AC |  | 38 | LC | NL |
| *Cacajao calvus* (I. Geoffroy, 1847) | | |  | AM |  | 10 | VU | NL |
| *Callicebus hoffmannsi* (Thomas, 1908) | | |  | PA |  | 34 | LC | NL |
| *Chiropotes satanas* (Hoffmannsegg, 1807) | | |  | PA |  | 44 | EN | CR |
| *Plecturocebus sp.* | | |  | AC |  | 38 | - | - |
| Cetacea | |  |  |  |  |  |  |  |
| Delphinidae | |  |  |  |  |  |  |  |
| *Sotalia fluviatilis* (Gervais, 1853) | | |  | AM |  | 52 | EN | NL |
| REPTILIA | |  |  |  |  |  |  |  |
| Crocodylia | |  |  |  |  |  |  |  |
| Alligatoridae | |  |  |  |  |  |  |  |
| *Caiman crocodilus* (Linnaeus, 1758) | | | PI | RO, PA, AC |  | 1, 14, 15, 30, 31, 38, 44, 49 | LC | NL |
| *Caiman latirostris* (Daudin, 1802) | | | PB, PE, BA |  |  | 29, 36 | LC | NL |
| *Caiman sp.* | | | BA |  |  | 57 | - | - |
| *Paleosuchus palpebrosus* (Cuvier, 1807) | | | CE |  |  | 31, 58 | LC | NL |
| *Melanosuchus niger* (Spix, 1825) | | |  | PA, AC |  | 32, 38, 44 | LC | NL |
| *Alligator sp.* | | |  | PA |  | 9, 54 | - | - |
| Squamata | |  |  |  |  |  |  |  |
| Teiidae | |  |  |  |  |  |  |  |
| *Salvator merianae* (Dumeril and Bibron, 1839) | | | RN, PB, PE, BA, CE | PA, TO | MG | 3,4,18,28,29,31,32,36,40,41,43,46,50,51,57,58,59,60 | LC | NL |
| *Tupinambis teguixin* (Linnaeus, 1758) | | |  | PA |  | 44, 54 | LC | NL |
| *Ameiva ameiva* (Linnaeus, 1758) | | | RN, CE |  |  | 31, 51 | LC | NL |
| Iguanidae | | |  |  |  |  |  |  |
| *Iguana iguana* (Linnaeus, 1758) | | | RN, PB, PE, BA, CE | AM, PA |  | 3, 4, 15, 22, 28, 29, 31, 32, 36, 40, 41, 43, 46, 50, 51, 57, 59 | LC | NL |
| Boidae | | |  |  |  |  |  |  |
| *Boa constrictor (*Linnaeus, 1758) | | | RN, PE, BA, CE | PA |  | 4, 18, 29, 31, 32, 46, 51, 57 | LC | NL |
| *Epicrates assisi* (Machado, 1945) | | | PE, BA, CE |  |  | 29, 31 | LC | NL |
| Epicrates sp. | | | BA |  |  | 57 | - | - |
| *Eunectes murinus* (Linnaeus, 1758) | | | BA, CE |  |  | 31, 57 | LC | NL |
| *Corallus hortulana* (Linnaeus, 1758) | | | CE |  |  | 31 | LC | NL |
| Viperidae | | |  |  |  |  |  |  |
| *Crotalus durissus* (Linnaeus, 1758) | | | PB, BA, CE |  |  | 31, 46, 57 | LC | NL |
| *Lachesis muta* (Linnaeus, 1766) | | | CE |  |  | 31 | LC | NL |
| B*othrops sp.* | | | BA |  |  | 57 | - | - |
| Phyllodactylidae | | |  |  |  |  |  |  |
| *Phyllopezus pollicaris* (Spix, 1825) | | | RN |  |  | 51 | LC | NL |
| Tropiduridae | | |  |  |  |  |  |  |
| *Tropidurus hispidus* (Spix, 1825) | | | RN |  |  | 51 | LC | NL |
| Colubridae | | |  |  |  |  |  |  |
| *Drymarchon corais* (Boié, 1827) | | | BA |  |  | 57 | LC | NL |
| Elapidae | | |  |  |  |  |  |  |
| *Micrurus ibiboboca* (Merren, 1820) | | | BA |  |  | 57 | DD | NL |
| Testudines | |  |  |  |  |  |  |  |
| Chelidae | |  |  |  |  |  |  |  |
| *Chelus fimbriatus* (Schneider, 1783) | | |  | AM, AC |  | 16, 52 | - | NL |
| *Mesoclemmys tuberculata* (Lüderwaldt, 1926) | | | RN, PB, CE |  |  | 31, 41, 50, 51, 59 | LC | NL |
| *Phrynops tuberosus* (Peters, 1870) | | | RN, CE |  |  | 31, 51 | - | NL |
| Testudinidae | | |  |  |  |  |  |  |
| *Chelonoidis sp.* | | |  | PA |  | 7, 11, 14, 32, 34 | - | - |
| *Chelonoidis denticulatus* (Linnaeus, 1766) | | |  | AM, PA, AC |  | 6, 9, 10, 12, 15, 16, 38, 44, 49, 52, 56 | VU | NL |
| *Chelonoidis carbonaria* (Spix, 1824) | | | BA, PB | AM, PA |  | 16, 31 40, 44, 49, 57 | - | NL |
| Podocnemididae | | |  |  |  |  |  |  |
| *Podocnemis unifilis* (Troschel, 1848) | | |  | AM, PA, AC |  | 6, 8, 9, 10, 12, 13, 14, 16, 32, 34, 38, 44, 49, 52 | VU | NL |
| *Peltocephalus dumerilianus* (Schweigger, 1812) | | |  | AM |  | 10, 12, 13, 14, 16 | VU | NL |
| *Podocnemis sextuberculata* (Cornalia, 1849) | | |  | AM |  | 9, 10, 12, 13, 14, 16, 52 | VU | NL |
| *Podocnemis erythrocephala* (Spix, 1824) | | |  | AM |  | 16 | VU | NL |
| *Podocnemis expansa* (Schweigger, 1812) | | |  | AM, PA, AC |  | 9, 10, 12, 13, 14, 16, 32, 38, 44, 49, 52 | LC | NL |
| Geoemydidae | | |  |  |  |  |  |  |
| *Rhinoclemmys punctularia* (Daudin, 1801) | | |  | AM, PA |  | 16, 54 | - | NL |
| Kinosternidae | | |  |  |  |  |  |  |
| *Kinosternon scorpioides* (Linnaeus, 1766) | | | CE |  |  | 31 | - | NL |
| Cheloniidae | | |  |  |  |  |  |  |
| *Chelonia mydas* (Linnaeus, 1758) | | | CE |  |  | 31 | EN | NL |
| *Eretmochelys imbricata* (Linnaeus, 1766) | | | CE |  |  | 31 | CR | NL |
| *Caretta caretta* (Linnaeus, 1758) | | | CE |  |  | 31 | VU | NL |
| *Lepidochelys olivacea* (Eschscholtz, 1829) | | | CE |  |  | 31 | EN | VU |
| Dermochelyidae | | |  |  |  |  |  |  |
| *Dermochelys coriacea* (Linnaeus, 1766) | | | CE |  |  | 31 | VU | NL |
| AMPHIBIOS | |  |  |  |  |  |  |  |
| Anura | |  |  |  |  |  |  |  |
| Leptodactylidae | |  |  |  |  |  |  |  |
| *Leptodactylus vastus* (A. Lutz, 1930) | | | PB |  |  | 31,46, 50, 59 | LC | NL |
| *Leptodactylus sp.* | | | CE |  |  | 57 | - | - |
| Ranidae | | |  |  |  |  |  |  |
| *Scinax x-signatus* (Spix, 1824) | | | CE |  |  | 57 | LC | NL |
| Bufonidae | | |  |  |  |  |  |  |
| *Rhinella jimi* (Stevaux, 2002) | | | CE |  |  | 31 | LC | LC |

Legends: Categories: MMA- Ministério do Meio Ambiente; DD- Data Deficient; LC - Least Concern; NT- Near Threatened; VU - Vulnerable; EN - Endangered; CR- Critical Endangered and NL-Not Listed. Regions of Brazil: NE (Northeast); N (North) e S (Southeast). Brazilian states: (AC = Acre; AL = Alagoas; AP = Amapá; AM = Amazonas; BA = Bahia; CE = Ceará; MA = Maranhão; MG = Minas Gerais; PA = Pará; PE = Pernambuco; PI = Piauí; PB = Paraíba; SE = Sergipe; TO = Tocantins; RN = Rio Grande do Norte; RO = Roraima. Note: the numbering of the reference lists (*) follows the additional file 1.
