# Supplementary material for: Use of wild vertebrates for consumption and bushmeat trade in Brazil: a review
Source: J Ethnobiol Ethnomed. 2023 Dec 19;19:64. doi: 10.1186/s13002-023-00628-x (PMC10729539; doi:10.1186/s13002-023-00628-x)
Supplement: Supplementary file 3 — Additional file 3. List of wild vertebrates species cited in articles on consumption and trade bushmeat and exclusive trade bushmeat. [file 13002_2023_628_MOESM3_ESM.docx]

**Additional file 2 –** List of wild vertebrates species cited in articles on consumption and trade bushmeat and exclusive trade bushmeat.

| Táxon/Species | | | Consumption and trade bushmeat | Region of citation/States Brazilian | | References* | Exclusive Trade bushmeat | Region of citation/States Brazilian | | References* | Status Conservation | |
| --- | --- | --- | --- | --- | --- | --- | --- | --- | --- | --- | --- | --- |
|  |  |  |  | NE | N |  |  | NE | N |  | IUCN (2021) | MMA (2022) |
| AVES |  | |  |  |  |  |  |  |  |  |  |  |
| Tinamiformes | |  |  |  |  |  |  |  |  |  |  |  |
| Tinamidae | |  |  |  |  |  |  |  |  |  |  |  |
| *Nothura boraquira* (Spix, 1825) | | |  |  |  |  | x | BA |  | 61 | LC | NL |
| *Crypturellus sp.* | | | x |  | AM | 6, 8 | x | BA |  | 61 | - | - |
| *Tinamus sp.* | | | x |  | AM | 6, 9 |  |  |  |  |  |  |
| Galliformes | |  |  |  |  |  |  |  |  |  |  |  |
| Cracidae | |  |  |  |  |  |  |  |  |  |  |  |
| *Mitu sp.* | | | x |  | AM | 9 |  |  |  |  | - | - |
| *Crax sp.* | | | x |  | AM | 6, 8, 9 |  |  |  |  | - | - |
| *Pauxi tuberosa* (Spix, 1825) | | | x |  | AM | 6, 10 |  |  |  |  | NT | NL |
| *Aburria sp.* | | | x |  | AM | 6 |  |  |  |  | - | - |
| *Penelope sp.* | | | x |  | AM | 8 |  |  |  |  | - | - |
| *Penelope jacquacu* (Spix, 1825) | | | x |  | AM | 6 |  |  |  |  | LC | NL |
| *Penelope superciliaris* (Temminck, 1815) | | | x | PI |  | 1 |  |  |  |  | NT | NL |
| Suliformes | |  |  |  |  |  |  |  |  |  |  |  |
| Anhingidae | |  |  |  |  |  |  |  |  |  |  |  |
| *Anhinga anhinga* (Linnaeus, 1766 | | | x |  | AM | 9 |  |  |  |  | LC | NL |
| Anseriformes | |  |  |  |  |  |  |  |  |  |  |  |
| Anatidae | |  |  |  |  |  |  |  |  |  |  |  |
| *Cairina moschata* (Linnaeus, 1758) | | | x |  | AM | 9 |  |  |  |  | LC | NL |
| Columbiformes | |  |  |  |  |  |  |  |  |  |  |  |
| Columbidae | |  |  |  |  |  |  |  |  |  |  |  |
| *Leptotila sp.* | | | x | AL |  | 23 |  |  |  |  | - | - |
| *Zenaida auriculata* (Des Murs, 1847) | | |  |  |  |  | x | BA |  | 61 | LC | NL |
| *Patagioenas picazuro* (Temminck, 1813) | | | x | AL |  | 23 |  |  |  |  | LC | NL |
| *Columbina picui* (Temminck, 1813) | | | x | AL |  | 23 | x | BA |  | 61 | LC | NL |
| *Columbina minuta* (Linnaeus, 1766) | | | x | AL |  | 23 |  |  |  |  | LC | NL |
| *Columbina talpacoti* (Temminck, 1810) | | | x | AL |  | 23 |  |  |  |  | LC | NL |
| *Columbina squammata* (Lesson, 1831) | | | x | AL |  | 23 |  |  |  |  | LC | NL |
| *Columbina sp.* | | |  |  |  |  | x | BA |  | 61 | - | - |
| MAMMALIA | |  |  |  |  |  |  |  |  |  |  |  |
| Artiodactyla | |  |  |  |  |  |  |  |  |  |  |  |
| Cervidae | |  |  |  |  |  |  |  |  |  |  |  |
| *Mazama sp*. | | | x |  | AM | 9, 10 | x | BA |  | 61 | - | - |
| *Mazama americana* (Erxleben, 1777) | | | x |  | AM | 6, 8 |  |  |  |  | DD | NL |
| *Subulo gouazoubira* (Fischer, 1824) | | | x | PI | AM | 1, 6, 8 |  |  |  |  | LC | NL |
| Tayassuidae | | |  |  |  |  |  |  |  |  |  |  |
| *Tayassu pecari* (Link, 1795) | | | x |  | AM | 6, 8, 9, 10 |  |  |  |  | VU | VU |
| *Pecari tajacu* (Linnaeus, 1758) | | | x | PI | AM | 1, 6, 8, 9, 10 | x |  | AM | 62 | LC | NL |
| Cingulata | |  |  |  |  |  |  |  |  |  |  |  |
| Chlamyphoridae | |  |  |  |  |  |  |  |  |  |  |  |
| *Priodontes maximus* (Kerr, 1792) | | | x |  | AM | 8 |  |  |  |  | VU | VU |
| *Euphractus sexcinctus* (Linnaeus 1758) | | | x | P1 |  | 1 | x | BA |  | 61 | LC | NL |
| Dasypodidae | | |  |  |  |  |  |  |  |  |  |  |
| *Dasypus sp.* | | | x |  | AM | 6, 8, 9 |  |  |  |  | - | - |
| *Dasypus novemcinctus* (Linnaeus, 1758) | | | x | PI |  | 1 | x | BA | PA | 61,63 | LC | NL |
| *Dasypus septemcinctus* (Linnaeus, 1758) | | | x | PI |  | 1 |  |  |  |  | LC | NL |
| *Dasypus beniensis* (Lönnberg, 1942) | | |  |  |  |  |  |  |  |  | - | NL |
| Rodentia | |  |  |  |  |  |  |  |  |  |  |  |
| Caviidae | | |  |  |  |  |  |  |  |  |  |  |
| *Cavia aperea* (Erxleben, 1777) | | |  |  |  |  | x | BA |  | 61 | LC | NL |
| *Hydrochoerus hydrochaeris* (Linnaeus, 1766) | | | x | P1 | AM | 1,6, 9 | x |  | PA | 63 | LC | NL |
| Erethizontidae | | |  |  |  |  |  |  |  |  |  |  |
| *Coendou bicolor* (Tschudi, 1844) | | | x |  | AM | 6 |  |  |  |  | LC | NL |
| *Coendou prehensilis* (Linnaeus, 1758) | | | x |  | AM | 6 |  |  |  |  | LC | NL |
| Cuniculidae | | |  |  |  |  |  |  |  |  |  |  |
| *Cuniculus paca* (Linnaeus, 1758) | | | x | PI | AM | 1, 6, 8, 9 ,10 | x |  | PA, AM | 62,63 | LC | NL |
| Dasyproctidae | | |  |  |  |  |  |  |  |  |  |  |
| *Dasyprocta fuliginosa* (Wagler, 1832) | | | x |  | AM | 6, 9, 10 |  |  |  |  | LC | NL |
| *Dasyprocta aguti* (Linnaeus, 1766) | | |  |  |  |  | x |  | PA | 63 | - | NL |
| *Dasyprocta prymnolopha* (Wagler, 1831) | | | x | PI |  | 1 |  |  |  |  | LC | NL |
| *Dasyprocta azarae* (Lichtenstein, 1823) | | |  |  |  |  |  |  |  |  | DD | NL |
| *Dasyprocta sp.* | | |  |  |  |  | x | BA |  | 61 | - | - |
| Perissodactyla | |  |  |  |  |  |  |  |  |  |  |  |
| Tapiridae | |  |  |  |  |  |  |  |  |  |  |  |
| *Tapirus terrestris* (Linnaeus, 1758) | | | x |  | AM | 6, 8, 10 | x |  | AM | 62 | VU | VU |
| Pilosa | |  |  |  |  |  |  |  |  |  |  |  |
| Myrmecophagidae | | |  |  |  |  |  |  |  |  |  |  |
| *Tamandua tetradactyla* (Linnaeus, 1758) | | | x | PI |  | 1 |  |  |  |  | LC | NL |
| Carnivora | |  |  |  |  |  |  |  |  |  |  |  |
| Mustelidae | | |  |  |  |  |  |  |  |  |  |  |
| *Eira barbara* (Linnaeus, 1758) | | | x |  | AM | 9 |  |  |  |  | LC | NL |
| Didelphimorphia | |  |  |  |  |  |  |  |  |  |  |  |
| Didelphidae | |  |  |  |  |  |  |  |  |  |  |  |
| *Didelphis marsupialis* (Linnaeus, 1758) | | | x |  | AM | 6 | x |  | PA | 63 | LC | NL |
| Sirenia | |  |  |  |  |  |  |  |  |  |  |  |
| Trichechidae | |  |  |  |  |  |  |  |  |  |  |  |
| *Trichechus inunguis* (Natterer, 1883) | | | x |  | AM | 9, 10 |  |  |  |  | VU | VU |
| Primates | |  |  |  |  |  |  |  |  |  |  |  |
| Atelidae | |  |  |  |  |  |  |  |  |  |  |  |
| *Alouatta juara* (Elliot, 1910) | | | x |  | AM | 10 |  |  |  |  | LC | NL |
| *Alouatta seniculus* (Linnaeus, 1766) | | | x |  | AM | 9 |  |  |  |  | LC | NL |
| *Lagothrix lagotricha* (Humboldt, 1812) | | | x |  | AM | 6, 8 |  |  |  |  | VU | NL |
| REPTILIA | |  |  |  |  |  |  |  |  |  |  |  |
| Crocodylia | |  |  |  |  |  |  |  |  |  |  |  |
| Alligatoridae | |  |  |  |  |  |  |  |  |  |  |  |
| *Caiman crocodilus* (Linnaeus, 1758) | | | x | PI | AM | 1, 9, 10 |  |  |  |  | LC | NL |
| *Caiman latirostris* (Daudin, 1802) | | |  |  |  |  | x |  | PA | 63 | LC | NL |
| *Melanosuchus niger* (Spix, 1825) | | |  |  |  |  | x |  | PA | 63 | LC | NL |
| Testudines | |  |  |  |  |  |  |  |  |  |  |  |
| Testudinidae | | |  |  |  |  |  |  |  |  |  |  |
| *Chelonoidis denticulatus* (Linnaeus, 1766) | | | x |  | AM | 6, 9, 10 |  |  |  |  | VU | NL |
| Podocnemididae | | |  |  |  |  |  |  |  |  |  |  |
| *Podocnemis unifilis* (Troschel, 1848) | | | x |  | AM | 6, 8, 9, 10 |  |  |  |  | VU | NL |
| *Peltocephalus dumerilianus* (Schweigger, 1812) | | | x |  | AM | 10 |  |  |  |  | VU | NL |
| *Podocnemis sextuberculata* (Cornalia, 1849) | | | x |  | AM | 9, 10 |  |  |  |  | VU | NL |
| *Podocnemis expansa* (Schweigger, 1812) | | | x |  | AM | 8, 9, 10 |  |  |  |  | LC | NL |

Legends: Categories: DD- Data Deficient; LC - Least Concern; NT- Near Threatened; VU- Vulnerable; EN- Endangered; CR- Critical Endangered and NL- Not Listed. Regions of Brazil: NE-Northeast; N-North. Brazilian states: AM - Amazonas; AL-Alagoas; BA-Bahia; PA-Pará, PI-Piauí. Note: the numbering of the reference lists (*) follows the additional file 1.
